# COVID-19-associated monocytic encephalitis (CAME): histological and proteomic evidence from autopsy

**DOI:** 10.1038/s41392-022-01291-6

**Published:** 2023-01-06

**Authors:** Pei-Pei Zhang, Zhi-Cheng He, Xiao-Hong Yao, Rui Tang, Jie Ma, Tao Luo, Chuhong Zhu, Tian-Ran Li, Xindong Liu, Dingyu Zhang, Shuyang Zhang, Yi-Fang Ping, Ling Leng, Xiu-Wu Bian

**Affiliations:** 1 Department of Pathology, the First Affiliated Hospital of University of Science & Technology of China, 230036 Hefei, Anhui P.R. China; 2 Institute of Pathology & Southwest Cancer Center, Southwest Hospital, Third Military Medical University (Army Medical University), and Key Laboratory of Tumor Immunopathology, Ministry of Education of China, 400038 Chongqing, P.R. China; 3 Department of Pathology, Ruijin Hospital, School of Medicine, Shanghai Jiaotong University, 200025, Shanghai, P.R. China; 4 Stem Cell and Regenerative Medicine Lab, Department of Medical Science Research Center, State Key Laboratory of Complex Severe and Rare Diseases, Translational Medicine Center, Peking Union Medical College Hospital, Peking Union Medical College and Chinese Academy of Medical Sciences, 100730 Beijing, P.R. China; 5 Department of Anatomy, Key Laboratory for Biomechanics and Tissue Engineering of Chongqing, State Key Laboratory of Trauma, Burn and Combined injury, Army Medical University (Third Military Medical University), 400038 Chongqing, P.R. China; 6 Wuhan Jinyintan Hospital (Wuhan Hospital for Infectious Diseases), 430015 Wuhan, Hubei P.R. China; 7 Department of Cardiology, Peking Union Medical College Hospital, Peking Union Medical College and Chinese Academy of Medical Sciences, 100730 Beijing, P.R. China

**Keywords:** Respiratory tract diseases, Infection

## Abstract

Severe neurological symptoms are associated with Coronavirus disease 2019 (COVID-19). However, the morphologic features, pathological nature and their potential mechanisms in patient brains have not been revealed despite evidence of neurotropic infection. In this study, neuropathological damages and infiltrating inflammatory cells were quantitatively evaluated by immunohistochemical staining, ultrastructural examination under electron microscopy, and an image threshold method, in postmortem brains from nine critically ill COVID-19 patients and nine age-matched cadavers of healthy individuals. Differentially expressed proteins were identified by quantitative proteomic assays. Histopathological findings included neurophagocytosis, microglia nodules, satellite phenomena, extensive edema, focal hemorrhage, and infarction, as well as infiltrating mononuclear cells. Immunostaining of COVID-19 brains revealed extensive activation of both microglia and astrocytes, severe damage of the blood–brain barrier (BBB) and various degrees of perivascular infiltration by predominantly CD14+/CD16+/CD141+/CCR7+/CD11c+ monocytes and occasionally CD4+/CD8+ T lymphocytes. Quantitative proteomic assays combined with bioinformatics analysis identified upregulated proteins predominantly involved in immune responses, autophagy and cellular metabolism in COVID-19 patient brains compared with control brains. Proteins involved in brain development, neuroprotection, and extracellular matrix proteins of the basement membrane were downregulated, potentially caused by the activation of transforming growth factor β receptor and vascular endothelial growth factor signaling pathways. Thus, our results define histopathological and molecular profiles of COVID-19-associated monocytic encephalitis (CAME) and suggest potential therapeutic targets.

## Introduction

Coronavirus disease 2019 (COVID-19), caused by severe acute respiratory syndrome coronavirus 2 (SARS-CoV-2), is defined as a systematic infectious disease resulting in multiple organ dysfunction syndromes (MODS).^1,2^ The global pandemic of this disease has caused more than 6.60 million deaths, especially in the elderly population with pre-existing pulmonary and cardiovascular diseases. Although most COVID-19 patients present primarily respiratory symptoms, a broad range of neurological symptoms, manifestations, and complications in patients with COVID-19 is increasingly recognized.^3–5^ The major neurological manifestations ranging from mild to severe symptoms include anxiety, depression, vision changes, impaired mobility, numbness in extremities, tremors, and myalgia, as well as loss of memory, hearing, taste, or smell.^2,6,7^ A range of persistent symptoms can remain after the onset of acute mild or severe COVID-19, known as “long COVID”, which can involve multiple organs including the central nervous system.^8–10^ Understanding the pathological nature and mechanisms of neurological manifestations is important for the improvement of COVID-19 outcomes.

Previous reports of neuropathological changes in the brains of COVID-19 patients include microglial nodules, neurophagocytosis, neuronal injury, and endotheliitis.^11–17^ These changes are recognized as non-specific, due to ischemia, hemorrhage, inflammation, and aging.^18–21^ It is also reported that the microvascular structure is damaged in COVID-19 patients.^17,22–24^ The levels of several cytokines such as interleukin (IL)-6, IL-8, IL-15, and macrophage inflammatory protein-1b (MIP-1b) were increased in a subset of patients and correlated with blood–brain barrier (BBB) disruption.^25^ Although post-mortem evidence supports the existence of encephalitis and/or meningoencephalitis in COVID-19 patients, a comprehensive analysis of COVID-19-associated brain inflammation with infiltrating immune cells has not been performed.^16,26,27^

Previous studies revealed possible infection by the SARS-CoV-2 virus in the brain tissues of COVID-19 patients.^1,21^ The neurotropism of the SARS-CoV-2 virus has been confirmed in mouse and organoid models.^28–31^ It is proposed that the SARS-CoV-2 virus enters the nervous system through the axonal or choroidal route.^28–30^ However, there is no correlation between the severity of neuropathological changes and the viral load.^7,13,29,32^ The detection of the SARS-CoV-2 virus in patients’ brain parenchyma, especially neurons and glial cells, is still controversial.^33,34^ It is noteworthy that severe neuropsychiatric manifestations did not match minor pathomorphological changes in the brain. Other mechanisms such as induced inflammatory and ischemic responses as well as other comorbidities may be involved in the pathogenesis of COVID-19 brain pathology.^33^ Previous observations of pan-encephalitis, “massive intracranial” and “diffuse petechial hemorrhage in the entire brain” in six patients with COVID-19 associated with T lymphocyte infiltration have been questioned.^11^ Better understanding of the molecular mechanisms underlying brain damage would be helpful for the development of therapeutics for this disease.

The contribution of viral (direct) damage and immunological (indirect) injury to the COVID-19 brain pathology needs to be clarified.^33,35^ To our knowledge, there is no comparison of COVID-19 brains with age-matched normal brains as controls. In this study, we defined the molecular features of COVID-19-associated encephalitis (CAME) with monocyte infiltration and glial cell activation based on systematic pathological and proteomic analyses of COVID-19 autopsy brains. Monocyte infiltration and microglial activation suggest their potential as therapeutic targets in COVID-19.

## Results

### Infiltration of monocytes with extensive microglial activation in COVID-19 brains

**Fig. 1:**
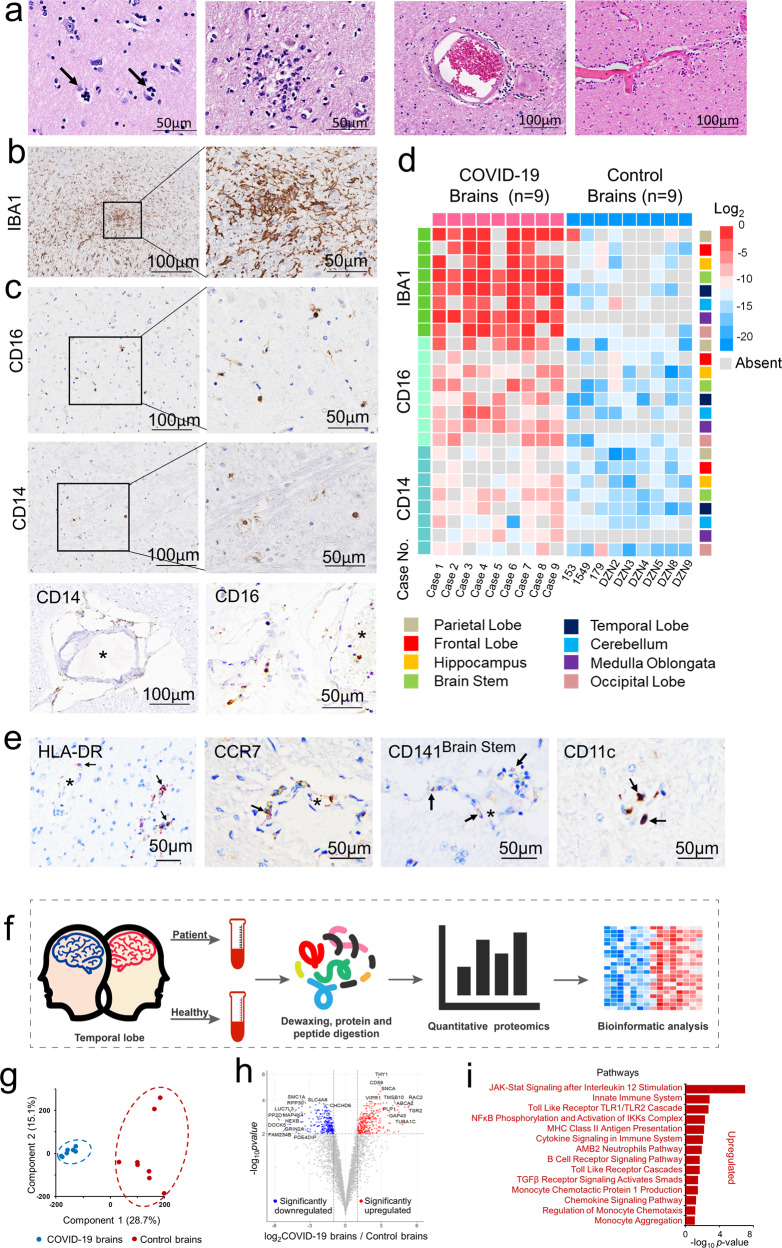
Monocyte infiltration and extensive microglia activation in COVID-19 brains. **a** Histology shows neuronophagia, microglial nodule and perivascular inflammation in COVID-19 brain tissues. **b** Immunohistochemical staining for IBA1 shows reactive activation of microglia. **c** Immunohistochemistry shows CD16+ and CD14+ inflammatory cells infiltrating perivascular space and parenchyma. **d** Quantitative analysis of the type and distribution of immune cells in the brains of all COVID-19 patients. In addition to CD16+ and CD14+ infiltrating cells, lymphocytic infiltration around the vessel is also seen (please see Supplementary Fig. S1). **e** The mononuclear cells (black arrows) in the microvessel (*) walls and perivascular spaces are positive for monocyte biomarkers HLA-DR, CCR7, CD141 and CD11c. **f** Scheme of proteomics study. **g** Principal component analysis of 15 samples based on quantitative profiles of proteins of brain tissues. Red and blue dots represent brain samples from COVID-19 patients and healthy controls. The red color represents control brains, while the blue color represents COVID-19 brains. **h** Volcano plots of the −log10 *p*-value vs. the log2 protein abundance comparisons between brains from normal subjects and those with COVID-19. Proteins outside the significance threshold lines (−log10 (*p*-value) > 2 and log2 (fold change) > 1 or <−1) are in red (upregulated) or blue (downregulated). The *p* values are calculated for proteins identified in brain tissues from the COVID-19 patients and healthy controls. **i** Proteomic analysis showing the activated monocyte-related pathway changes and release of inflammatory factors

To examine inflammatory features, histopathological examination of brain specimens derived from nine deceased COVID-19 patients (aged from 57 to 87 years old, median age 77) and nine control brains from normal donors (aged from 50 to 70 years old, median age 58) was performed (Table 1 and Supplementary Table S1). Neuronophagia (9/9 cases) and microglial nodules (5/9 cases) were found (Fig. 1a), although neither SARS-CoV-2 mRNA nor viral proteins were detectable in neurons and glial cells in the brains of these patients with confirmed presence of SARS-CoV-2 in their lungs.^1^ The infiltrating inflammatory mononuclear cells accumulated in the perivascular space and brain parenchyma (9/9 cases) (Fig. 1a). In contrast, neither neuronophagia nor microglial nodules were found in control brains. Microglia with enlarged cell soma and thickened processes were significantly increased in COVID-19 brains compared with healthy control brains as revealed by ionized calcium-binding adaptor molecule 1 (IBA1) immunostaining (Fig. 1b), suggesting increased microglial activation in COVID-19 brains. Immunostaining showed that the infiltrating cells were predominantly CD14^+^ and CD16^+^ monocytes, with fewer CD3^+^, CD4^+^, CD8^+^ and CD20^+^ lymphocytes (Fig. 1c and Supplementary Fig. S1). Quantitative analysis indicated significant increases in IBA-1+ microglia, CD16+ and CD14+ mononuclear cells in COVID-19 patient brains when compared with control brains. The increases in inflammatory cells were predominantly found in perivascular space (Robin–Virchow space) and parenchyma (especially around necrotic neurons, such as neuronophagia) of the temporal lobe, frontal lobe, brain stem and medulla oblongata (Fig. 1d). Most infiltrating CD16+ and CD14+ mononuclear cells expressed higher levels of HLA-DR, CCR7, CD141, and CD11c, suggesting the nature of monocytes (Fig. 1e). IBA-1+ microglia with longer processes also showed stronger staining for CD11c in COVID-19 brains when compared with controls. Therefore, our results provide evidence of the presence of COVID-19-associated brain inflammation, characterized by monocyte-dominant encephalitis.

**Table 1:** Clinical information of COVID-19 patients

	Case 1	Case 2	Case 3	Case 4	Case 5	Case 6	Case 7	Case 8	Case 9
Sex	M	M	F	F	F	M	M	F	F
Age	77	87	57	87	84	59	68	80	59
Course (days)	18	11	29	38	21	55	52	62	49
Medical history	DM	Hypertension CAHD	Healthy	Healthy	Hypertension, Cavavar cerebral infarction, Brain atrophy, Atherosclerosis	DM	DM	DM	Hypertension DM
Initial and subsequent symptoms	Fever, chivering, progressive expiratory dyspnea	Asthma, cough, both lower limbs scattered in spots	Fever, cough, anorexia, diarrhea	Fever, cough, pain in the throat, oppression in the chest, dyspnea	Fever, dry cough, body muscle soreness, vomiting	Fever, cough, oppression in the chest	Fever, cough, wheezy, bad appetite	Fever, cough	Fever, cough, oppression in chest
Neurological symptoms	Disturbance of consciousness, coma	Disturbance of consciousness, restlessness deep coma	Disturbance of consciousness, out of spirit, abnormal gait, deep coma	Coma	Out of spirit, abnormal gait, mild disturbance of consciousness, headache	Out of spirit, tension and anxiety, deep coma	Out of spirit, abnormal gait, deep coma	Out of spirit, abnormal gait, disturbance of consciousness, deep coma	Out of spirit, abnormal gait
Bacterial infection (culture)	*Klebsiella* (blood)	No (blood)	*A. baumannii*, *P. aeruginosa*, *E. coli, Klebsiella* (blood)	No (blood)	No (blood)	*A. baumannii*, *P. aeruginosa*, *Klebsiella* (sputum)	No (blood)	*P. aeruginosa* *A. baumannii* (BALF)	No (blood)
Main diagnoses and the causes of death	1. COVID-19 (critical) 2. Respiratory failure 3. Renal insufficiency	1. COVID19 (critical) 2. Septic shock 3. Respiratory failure 4. Lacunar cerebral infarction	1. COVID-19 (critical) 2. Septic shock 3. ARDS 4. Bacterial pneumonia 5. Massive airway hemorrhage	1. COVID-19 (critical) 2. Respiratory failure 3. Heart failure	1. COVID-19 (critical) 2. Renal insufficiency 3. Bacterial pneumonia 4. Respiratory failure 5. Multiple cerebral infarction	1. COVID-19 (critical) 2. ARDS 3. Bacterial pneumonia 4. Renal insufficiency	1. COVID-19 (critical) 2. Septic shock 3. ARDS	1. COVID-19 (critical) 2. Lung cancer 3. Lacunar cerebral infarction 4. Bacterial pneumonia 5. ARDS	1. COVID-19 (critical) 2. ARDS 3. Septic shock 4. Renal insufficiency

*CAHD* coronary atherosclerotic heart disease, *DM* diabetes mellitus, *COPD* chronic obstructive pulmonary disease, *ARDS* acute respiratory distress syndrome, *BALF* bronchoalveolar lavage fluid, *ESBLs* extended-spectrum β-lactamases

To investigate the neuroinflammatory pathways in COVID-19-associated monocytic encephalitis, we compared proteomic profiles between COVID-19 and control brains (Fig. 1f). A total of 4587 proteins were identified in brain tissues from COVID-19 patients and 4414 proteins from control brains. There were 4351 proteins (93.6%) detected in both COVID-19 and control brains. Principal component analysis (PCA) was used to quantitate the dissimilarity of the proteins identified in the COVID-19 and the control brains. The results revealed significant differences between these two groups (Fig. 1g) with a total of 572 proteins differentially expressed (Benjamini-Hochberg adjusted *p* < 0.01) in COVID-19 brains compared to control brains (Supplementary Table S2). Among these, 294 proteins were upregulated (COVID-19/Control > 2 fold and Benjamini-Hochberg adjusted *p* < 0.01) and 278 proteins were downregulated (COVID-19/Control < 1/2 fold and *p* < 0.01) in the COVID-19 brain tissues (Fig. 1h). Proteomic analysis showed changes in signal pathways including JAK-STAT signaling, toll-like receptor TLR1/TLR2 cascade, NFκB phosphorylation, activation of IKKs complex and MHC class II antigen presentation in the COVID-19 brains. The proteins associated with immune signal pathways that drive the release of inflammatory factors, such as JAK-STAT signaling after interleukin (IL)-12 stimulation (PITPNA, CDC42, RAP1B, CFL1, RPLP0, SNRPA1, SOD2, RALA, PPIA), monocyte chemotactic protein 1 (HMGB1, APOD) production were upregulated in the COVID-19 brains (Fig. 1i). Proteomics results indicate that microglia and astrocytes in the brains of COVID-19 patients are activated to release inflammatory mediators (IL-4, -6, -12, etc.) causing brain damage.

Leptomeningitis was found in the brains of three COVID-19 patients with severe secondary pulmonary bacterial infections and sepsis (Table 1, Supplementary Fig. S2a). The exudated inflammatory cells were primarily CD14^+^ and CD16^+^ monocytes (Supplementary Fig. S2b). In addition, immunostaining detected marked GFAP-positive hyperplasia of astrocytes in COVID-19 brains (Supplementary Fig. S1), implying their extensive activation and possible participation in brain injury.

### Structural disruption and molecular alterations of the blood–brain barrier in COVID-19 brains

**Fig. 2:**
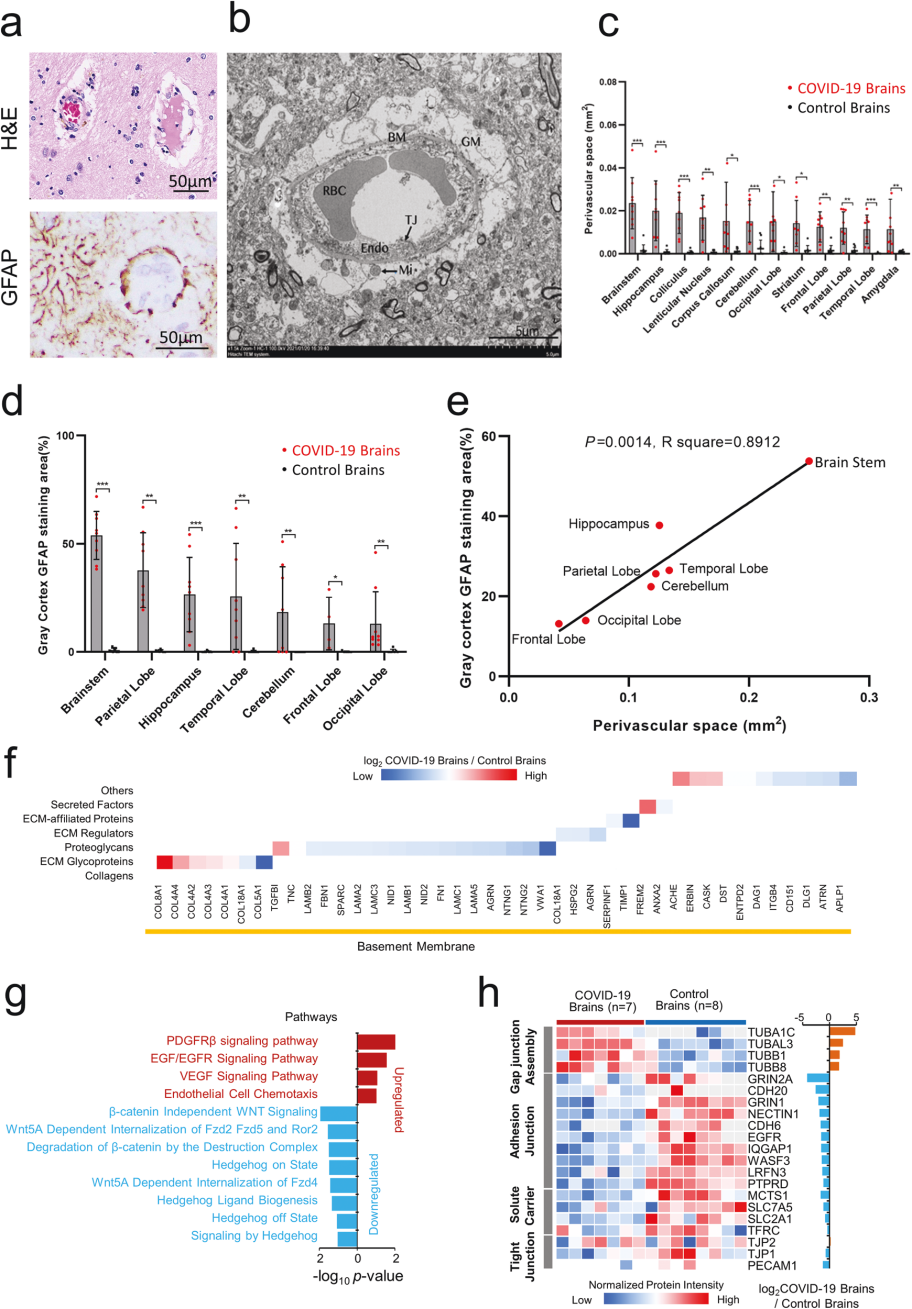
Structural damage and molecular alterations of blood–brain barrier (BBB) in COVID-19 brains. **a** Hyperemia and dilatation of blood vessels, perivascular infiltration of inflammatory cells, destruction of glial membrane and damage of BBB. GFAP immunohistochemistry showing damaged and discontinuous glial membranes were damaged and discontinuous. **b** Damaged BBB with degenerative capillary endothelial cells (Endo), abnormal tight junction, broken basement membranes (BM) and swollen glial membrane (GM, i.e., astrocytic end-feet). Organelles such as mitochondria (Mi) show degeneration. RBC, red blood cell. **c** Quantitative analysis of proteins significantly increased in COVID-19 patients correlated with the degree of cerebral edema. **d** Elevated glial fibrillary acidic protein (GFAP) is seen in the brain of COVID-19 patients and is associated with glial membrane destruction and astrocyte activation. **e** Perivascular space correlated with astrocyte activation. **f** Heatmap analysis of six types of ECM categories identified in the basement membrane (BM) according to log2 fold changes of COVID-19 brains vs. control brains. Red and blue boxes indicate proteins with increased or decreased abundance, respectively, in brain tissues from COVID-19 patients. **g** Functional pathway enrichment analysis of differentially expressed proteins in brain tissues from COVID-19 patients vs. healthy controls. Color bars represent the −log10 *P* value of the upregulated or downregulated functional categories with COVID-19 brains vs. control brains. **h** Functional analysis of differentially expressed proteins in brain tissues from patients with COVID-19 vs. controls. Columns on the left of the heatmap represent different function categories. The right of the heatmap represents gene names. Red and blue boxes indicate the log10 *p*-value of the intensity of the enriched or depleted proteins, respectively. The histogram shows the ratio of protein intensities from COVID-19 brains compared to control brains. *Y*-axis represents the log_2_ COVID-19/control brains

To better understand the inflammatory exudation in COVID-19 brains, we investigated the pathological changes of blood–brain barrier (BBB). We found swelling endothelial cells and discontinuous perivascular astrocytic end-feet layers with edema (Fig. 2a). Electron microscopic examination confirmed BBB damage with capillary endothelial cell swelling, abnormal tight junctions, broken basement, and glial membrane swelling (Fig. 2b). Quantitative analysis of vasogenic brain edema in the whole brain showed that the enlargement of perivascular space in the brainstem was most severe among various brain regions (Fig. 2c, d). In addition, the degree of brain edema was significantly correlated with BBB damage and the activation of astrocytes (Fig. 2e). Furthermore, brain endothelial cells were sporadically positive for SARS-CoV-2 protein (Supplementary Fig. S3a). These results indicate disrupted BBB integrity in COVID-19 brains and viral infection in the vasculature.

Proteomics analysis showed that extracellular matrix proteins, especially most components of the basement membrane (BM) were down-regulated in COVID-19 brain tissues as compared with control brains. The most changed components of BM included laminins (LAMB2, LAMA2, LAMC3, LAMB1, LAMC1, and LAMA5) and proteoglycans (COL18A1, HSPG2, and AGRN), with some BM specific collagens (IV, VIII, XVIII, and V collagens) remaining in COVID-19 brains, as compared with the control brains (Fig. 2f). After excluding COVID-19 and control brains because of low quality, seven COVID-19 and eight control brains were used in the comparative proteomic analyses. Signaling pathway analysis in COVID-19 brain tissues showed that PDGFR-β, EGF/EGFR and VEGF pathways were upregulated, while Wnt and hedgehog signaling pathways were downregulated (Fig. 2g), resulting in the abnormality in BBB-associated molecules (Fig. 2h) which physically maintain BBB integrity and function by forming adhesion junctions, solute carriers, tight junctions, efflux pumps, material transport and vesicle-mediated transport.^36,37^ Thus, our results reveal protein profiles associated with BBB pathogenesis, which might exacerbate COVID-19-associated encephalitis.

### COVID-19-associated encephalitis similarities in cytokine profiles and between SARS-CoV2-mediated lung and liver injury

**Fig. 3:**
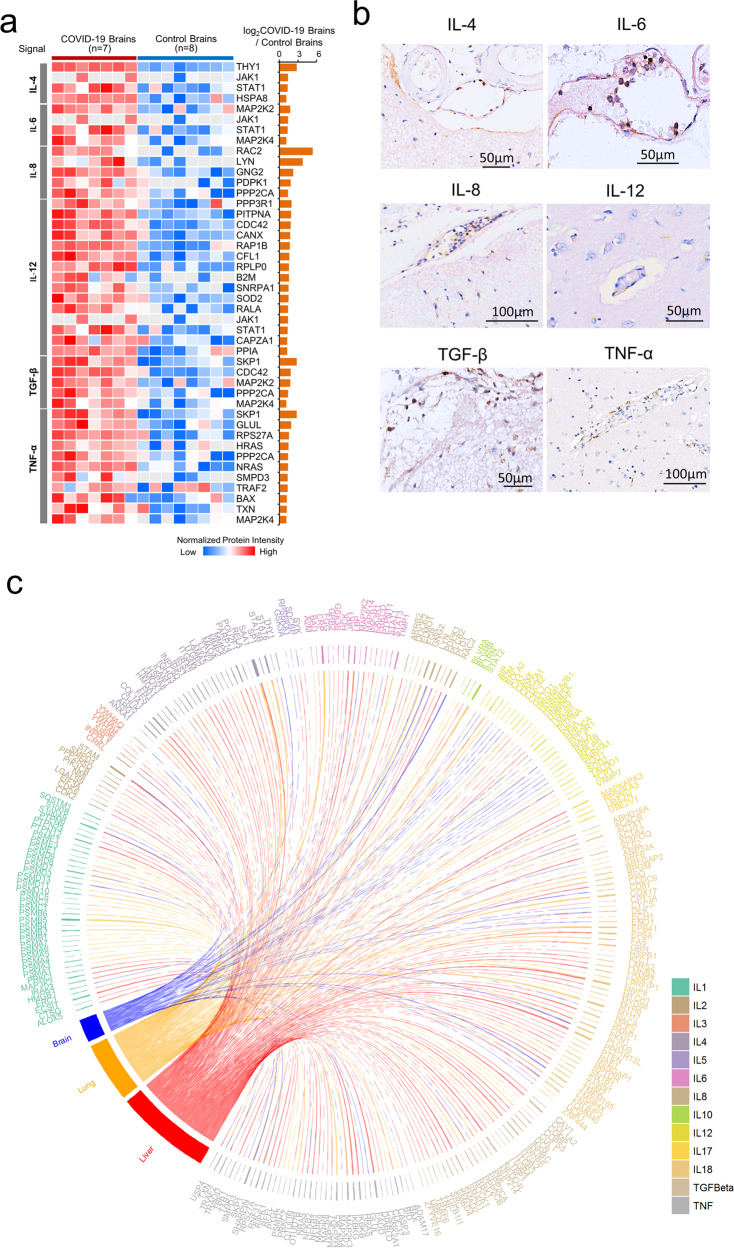
Similar cytokine profiles in COVID-19-associated encephalitis and SARS-CoV2-induced lung and liver injury. **a** Inflammation analysis of differentially expressed proteins in brain tissues from patients diagnosed with COVID-19 vs. controls. Columns on the left of the heatmap represent different inflammatory factors. The right of the heatmap lists gene names. Red and blue boxes indicate normalized intensity of the enriched or depleted proteins, respectively. Histogram shows the ratio of protein intensities from the COVID-19 patients. *Y*-axis represents the log_2_ COVID-19/Controls. **b** Immunohistochemistry shows endothelial cells, lymphocytes, and monocytes positive for IL-4, IL-6, IL-8, IL-12, TGF- β, and TNF-α. **c** The similarities of inflammatory factors in the brain, lung, and liver tissues of COVID-19 patients

For a better understanding of the molecular basis for inflammatory neuropathology, we analyzed inflammatory mediators associated with encephalitis. We found that the levels of IL-4, IL-6, IL-8, IL-12, TGF-β, and TNF-ɑ related pathways were upregulated in COVID-19 brains compared with control brains (Fig. 3a). We verified cytokine expression by immunohistochemical staining which showed that these cytokines were mainly expressed on vascular endothelial cells, monocytes, lymphocytes, and microglia (Fig. 3b). Among the cytokines detected, IL-6 was significantly also increased in the peripheral blood of COVID-19 patients (*p* < 0.05), suggesting that IL-6 may associate with encephalitis (Supplementary Fig. S3b). We previously showed inflammatory responses and cytokine production in the lung and liver tissues of COVID-19 patients as important pathological features of SARS-CoV-2-infected human tissues.^36,37^ We thus compared differentially expressed cytokines associated with inflammatory responses between COVID-19 patients and controls. We found that the brain, lung, and liver tissues from COVID-19 patients contained increased IL-1, IL-8, IL-12, IL-18, and TNF-α. These results suggest that proinflammatory cytokines may participate in the formation of the systemic storm (Fig. 3c).

### Inflammation-associated ischemic neuropathology in COVID-19 brains

**Fig. 4:**
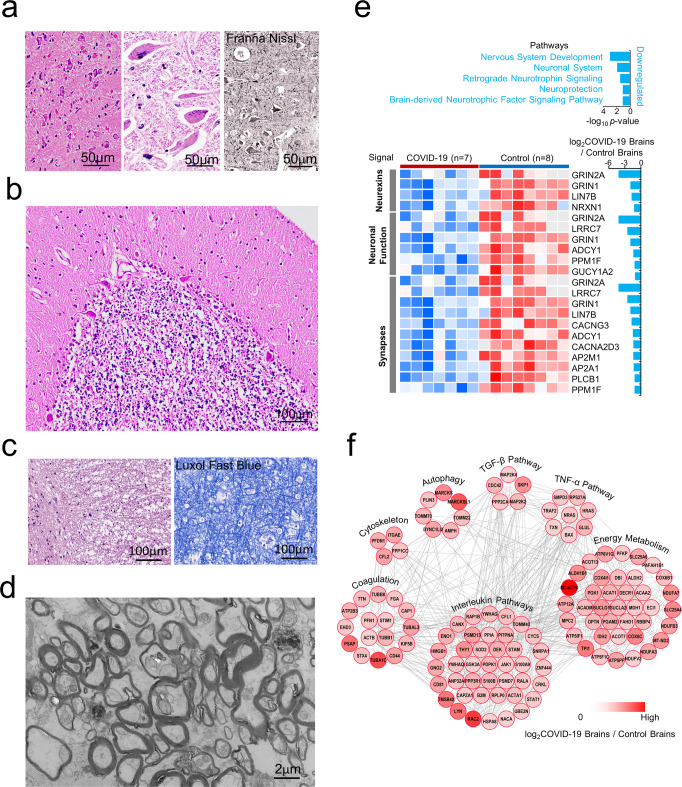
Neuron and myelin sheath damage associated with abnormal protein expression in COVID-19 brains. **a** Cerebral neurons show various degrees of damage, including red degeneration of neurons, dissolution and disappearance of Nissl bodies, and formation of giant neurons in gray matter. **b** The number of cerebellar Purkinje and granular cells was decreased and their dendrites and axons show injured. **c** Loose and rupture of white matter. **d** Electron microscopic examination shows the swollen axons and myelin sheath (scale bars 2 μm). **e** Functional pathway enrichment analysis of differentially expressed proteins in brain tissues from patients with COVID-19 vs. controls. Blue bars represent the −log_10_ *p*-value of the downregulated functional categories with COVID-19 vs. controls. Columns on the left of the heatmap represent different function categories. The right of the heatmap represents the gene names. Red and blue boxes indicate normalized intensity of the enriched or depleted proteins, respectively. Histogram showing the ratio of protein intensities from COVID-19 brains compared to control brains. *Y*-axis represents the log_2_ COVID-19/controls. **f** Interaction network of proteins differentially expressed in brains of patients with COVID-19 vs. controls. The primary biological process analyses include a map of functional categories. Red circles represent proteins with high expression in brain tissue from patients diagnosed with COVID-19 compared to controls. Color gradient indicates the protein abundance levels in the brains of COVID-19 patients

In addition to inflammatory cell infiltration and BBB damage, we found non-inflammatory neuropathology in COVID-19 brains. Red degeneration of neurons, dissolution and disappearance of Nissl bodies, and formation of giant neurons were found in the gray matter (Fig. 4a) with decreased number of cerebellar Purkinje and granular cells (Fig. 4b) in COVID-19 brains. In the white matter, the myelin sheath was swollen, broken, or lost as revealed by electron microscopy and Luxol fast blue staining, a special dye for myelin (Fig. 4c, d). Tissues from five of nine COVID-19 patient brains showed multicystic encephalomalacia.

Signal pathway analysis of COVID-19 brains showed that elements of nervous system development, retrograde neurotrophin signaling, neuroprotection and brain-derived neurotrophic factor signaling pathways were downregulated (Fig. 4e).^38,39^ Multiple molecules and pathways as well as protein–protein interactions were involved in COVID-19 neuropathology (Fig. 4f). Most of the proteins for autophagy, which recycles damaged organelles in response to nutrient deprivation, oxygen depletion and viral infection were upregulated in COVID-19 brains.^36–38^ Energy metabolism-associated proteins were also highly enriched in COVID-19 brains, including glycometabolism (TPI1, ALDH1B1, ALDH2, PGK1, etc.) and oxidative phosphorylation (MT-ATP8, COX6C, COX4I1, SLC25A1, NDUFA7, etc.) related proteins. These results suggest that the autophagy process might be responsible for the high-level energy metabolism in COVID-19 brains.

We additionally found focal or massive cerebral hemorrhages in tissues from six COVID-19 patient’s brains. Massive hemorrhage was seen under the pia mater of the cerebellum (Supplementary Fig. S2c). Multiple hemorrhagic areas were frequently visualized in the frontal and parietal lobes of COVID-19 brains (Supplementary Fig. S2d). Mixed thrombus and hyaline thrombus were seen in six patient brains (Supplementary Fig. S2e, f). One patient brain contained niches of leukocyte aggregation in multiple blood vessels (Supplementary Fig. S2g). Necrotic vasculitis was detected in the brains of two patients (Supplementary Fig. S2h).

## Discussion

In this study, we defined the neuropathological nature in the brain as COVID-19-associated monocytic encephalitis (CAME), which might be caused jointly by aseptic perivascular and parenchymal inflammation, SARS-CoV-2 infection of endothelial cells, hypoxic ischemia encephalopathy, and secondary infection of microorganisms in the lung.^1,11–14^ During the past 2 years, several studies reported COVID-19-associated damage in the brain. However, the existence and the cause of encephalitis/meningitis have been in dispute. The previous reports on the diagnosis of meningitis and/or encephalitis in COVID-19 depended on clinical manifestations such as neck stiffness and vomiting, images of CT and MRI, and PCR detection for viral RNA in cerebrospinal fluid.^26^ There was no evidence of pathology until the COVID-19 autopsy revealed primitive neuropathological findings in the brain.^16^ The controversial results of virus detection and the unknown nature of inflammatory pathology promoted us to further study the mechanism.^15–21^ Our current results reveal the pathological nature of CAME with monocyte-dominant encephalitis and microglial activation-mediated inflammatory damage.

Our study provides the first spatial proteomic basis of CAME pathogenesis. Although encephalitis/meningitis has been reported in COVID-19 patients, there has been no molecular analysis of the relationship between cytokine expression and neuropathological changes in the inflammatory responses of glia in the brain.^40–42^ Previous observations of COVID-19 brains lacked not only adequate (normal) control brains but also comparative molecular analysis.^32^ In this study, we found that the infiltrating inflammatory cells were predominantly monocytes in the perivascular sheath and parenchyma, in addition to a minority of T lymphocytes. Proteomics analysis showed activation of inflammation pathways including JAK-STAT signaling, TLR1/TLR2 cascade, NFκB phosphorylation and IKK complex, as well as other innate immunity components that support the monocytic inflammation nature of the encephalitis.

This study links inflammatory cell infiltration, glial cell activation, BBB disruption and proteomic changes in CAME. We analyzed the inflammatory characteristics of COVID-19 brains and found that all nine COVID-19 patient brains contained various degrees of encephalitis-related pathological changes with activation of microglia and astrocytes, as well as BBB damage, characterized by perivascular and CD14+/CD16+ monocyte infiltration. These monocytes expressed high levels of CD141, CCR7 and CD11c, suggesting their activation in the perivascular space and parenchyma. Activation of monocytes and microglia is likely to damage endothelial cells, pericytes, and glial membrane resulting in the disruption of BBB through the release of inflammatory mediators.^40^ Our results thus indicate that IBA1+/CD11c+ microglia and infiltrating monocytes contribute to inflammatory damage of the brain.

Activation of microglia and astrocytes was found in diverse brain regions of COVID-19 patients.^20,43,44^ Astrocytes and microglia play important roles in maintaining brain function. Astrocytic processes participate in the formation of BBB by their end feet. In CAME, activated astrocytes were mainly located in subpial gray matter and around blood vessels, preferentially in the brainstem, followed by the parietal lobe, hippocampus, and temporal lobe. Like astrocytes, microglial activation also mainly occurred in the brainstem and parietal lobe. There was a positive correlation between the degree of brain edema in COVID-19 patients and the activation of astrocytes. The swollen astrocytes and discontinuous end feet resulted in BBB dysfunction. Moreover, the number of activated astrocytes was also positively associated with more severe neuropathological changes and survival time of COVID-19 patients. Our study indicates activated signaling pathways associated with elevated inflammatory mediators linked to the innate immunity of microglia and astrocytes in COVID-19 brains. Excessive production of inflammatory cytokines such as IL-12 was associated with tissue damage and activated microglia may disrupt BBB. Thus, monocyte-mediated over-activation of the innate immunity arm may be an important mechanistic basis for COVID-19 neuropathology, in which infiltrating monocytes and resident microglia might be of therapeutic significance in the management of COVID-19 brain injury.

Despite the significant progress in genomic profiling of COVID-19 patients, and reports of viral detection in autopsy brains and cerebrospinal fluid, we did not find SARS-CoV-2 in patient brains by PCR and the viral proteins were not seen in neurons and glial cells by immunostaining.^31,40,45^ There is no evidence of the virus particles in neurons. However, we detected viral protein in microvascular endothelial cells and monocytes within the vascular lumens in patient brains, which might be the reason for positive detection by PCR in previous reports. Ischemia and inflammatory cytokines may cause damage to BBB due to the pathogenesis of the basement membrane, cellular junctions, and glial membrane (astrocytic end-feet). In addition, neuronal, axonal and myelin injuries as well as infarction were found in COVID-19 brains, probably due to microthrombus, vasculitis and cytokines.

Our cohort study found that leptomeningitis complicated with encephalitis could be seen in some critically ill COVID-19 patients. The infectious leptomeningitis was linked to the systematic spread of bacteria from the lung. Our study identified three cases of leptomeningitis related to pulmonary infection by bacteria (*Pseudomonas aeruginosa*, *Acinetobacter baumannii*, *Escherichia coli* and *Klebsiella pneumoniae*) (Table 1). These patients also suffered from septic shock in which inflammatory factors from the lung perfused the brain and activated microglia to induce reactive hyperplasia of neurotoxic astrocytes, resulting in irreversible brain damage. Neuronal, axonal and myelin injuries, as well as infarction, were found in COVID-19 brains probably due to microthrombus, vasculitis and cytokines.

The elderly are more susceptible to COVID-19 with a critical illness.^3,5^ In our study, we found amyloid-β accumulation in the brain of one COVID-19 patient with Alzheimer’s disease. It has been reported that patients with COVID-19 often have neurological complications, such as Alzheimer’s and Parkinson’s diseases, which call for further studies to discover novel methods for clinical detection and intervention of adverse reactions.^46,47^

Our study was performed on the brains of deceased COVID-19 patients with SARS-CoV-2 infection in 2020 in Wuhan, China. The limitations of this study include a lack of experiments from animal models, in vitro organoid infection, and protein functions to substantiate the conclusions. Also, further investigation should include a comparison of brain pathology and protein profiling changes with Omicron-infected COVID-19 patients.

## Materials and methods

### Autopsy and brains

COVID-19 autopsy was conducted at 8–24 h after the death of the patients in Wuhan, China from February 18 to April 4, 2020.^1^ COVID-19 brain samples from nine autopsy cases with critically ill COVID-19 and control brain samples from nine age-matched healthy cadavers without neurological diseases were obtained from the Biobank of Southwest Hospital, Third Military Medical University (TMMU), with informed written consent. The study was conducted in accordance with regulations issued by the National Health Commission of China and the Helsinki Declaration.

### Preparation for histopathologic examination

Pre-mounted, 3 µm-thick serial sections of 10% neutral formalin-fixed paraffin-embedded brain tissues from 14 regions (frontal lobe, parietal lobe, temporal lobe, occipital lobe, hippocampus, striatum, brainstem, amygdala, thalamus, lenticular nucleus, cerebellum, corpus callosum, olfactory nerve, medulla oblongata) of COVID-19 cases (*n* = 9) and control brains (*n* = 9) were stained with haematoxylin and eosin (H&E).

### Quantitative analysis of immunohistochemical staining

Formalin-fixed, paraffin-embedded brain specimens from each region were used for immunohistochemistry (IHC). Standard Leica Bond protocol for IHC was used and all immunohistochemical markers used in this article are detailed in Supplementary Table S3. The primary antibodies included IBA-1 (NBP1-83077, 1:1000 dilution, Novus Biologicals, USA), CD3 (Kit-0003, ready for use, MXB Biotechnology, China), CD4 (RMA-0620, ready for use, MXB Biotechnology), CD8 (MAB-0021, ready for use, MXB Biotechnology), CD14 (ab181470, 1:250 dilution, Abcam, USA), CD16 (ab46679, 1:250 dilution, Abcam), CD141(ab109189, Abcam, USA), CD11c (PA5-35326, ThermoFisher, USA), HLA-DR (ab92511, Abcam, USA), CCR7 (ab253187, Abcam, USA), SARS-CoV-2 spike protein (40150-T62-COV2, 1:1000 dilution, Sino Biological Inc., China), GFAP (18953-S, 1:400 dilution, IBL America, USA), IL-6 (ab6672, 1:250, Abcam), IL-4 (ab239508, 1:400 dilution, Abcam), IL-8 (AHP781, 1:1000 dilution, Bio-Rad, Germany), IL-12(ab9992, 1:1200 dilution, Abcam), TGF-β (21898-1-AP, 1:1000 dilution, Proteintech, USA), and TNF-α (8184S, 1:20 dilution, Cell Signaling Technology, USA). The Leica Bond Polymer Refine DAB detection kit was used according to the manufacturer’s instructions. Immunohistochemical staining was done on the Ventana BenchMark ULTRA IHC (Roche Diagnostics, Basel, Switzerland). Positive and negative controls were included in each run.

Digital section images of immunohistochemically stained slides were obtained using an Aperio Digital Pathology Slide Scanner (Aperio GT 450 DX). The obtained high-resolution digital images were managed using the Leica Image Scope software (version 12.4.0.5043). For further evaluation, at least five regions (1 mm x 1 mm) were selected on each of the selected images of CD3, CD4, CD8, CD14, CD16, CD20, IBA-1 and GFAP immunohostochemically stained slides. The selected microscopic image areas were decomposed into isolated individual stains (the red, green, blue OD color vectors) by color decovolution algorithm, and were separated by thresholding the HSV images.

The percentages of IHC-positive areas in the whole selected images were calculated. Heatmap was performed by the R package (version 4.1.1), which represented the log2 mean value of each marker after normalization. To measure the severity of perivascular space enlargement, the areas occupied by enlarged perivascular spaces were semi-automatically segmented (Supplemental Materials).

### Transmission electron microscopy (TEM)

Samples for TEM were processed according to standard procedures.^1^ Fresh sample from all cases was fixed in 2.5% glutaraldehyde in 0.1 M phosphoric buffer (pH 7.4) for 24 h, post-fixed with 1% osmium tetroxide and dehydrated with gradient alcohol. Tissues were then embedded in Eponate 12™ Kit with DMP-30 (18010, TED PELLA Inc.). Suitable sections (100 nm thick) were identified by double-staining with uranium acetate and lead citramalic acid, respectively. Thin sections were examined using a Hitachi HT7700 transmission electron microscope.

### Quantitative reverse-transcription polymerase chain reaction (qRT-PCR) and digital droplet PCR (ddPCR)

qRT-PCR and ddPCR were performed according to the standard procedures as described previously.^1^ Sequence of the primers and probes for the SARS-Cov-2 virus was obtained from National Institute for Viral Disease Control and Prevention (http://nmdc.cn/#/nCoV). Detailed information on sequences was summarized in Supplementary Table S4. Total RNA was extracted from FFPF samples by an FFPF RNA Kit (ADx-FF04, Amoy Diagnostics Co., Ltd, China). Nucleic acid extraction of SARS-Cov-2 was used by real-time RT-PCR method with a SARS-Cov-2 Nucleic Acid Detection Kit (8.0131901X024E, Amoy Diagnostics Co., Ltd) according to the manufacturer’s instructions. Digital droplet PCR assays were performed on QX200 AutoDG Droplet Digital PCR system (Bio-Rad) with One-Step RT-ddPCR Advanced Kit (186-4021, Bio-Rad Co., Ltd).

### Proteomics data acquisition

Pool peptides were fractioned by using high-pH HPLC to reduce sample complexity (Supplemental Materials). To acquire mass spectrometry (MS) data, the data-independent acquisition (DIA) scan mode was used for single-shot samples, whereas the fractionated samples of the pool were acquired with a top 40 data-dependent acquisition (DDA) scan mode. Both acquisition schemes were combined with the same liquid chromatography gradient. The MS data of the fractionated pool (DDA MS data, 24 fractions) and the single-shot samples (DIA MS data) were used to generate a DDA-library and direct-DIA-library, respectively. All searches were performed using the human Swiss-Prot protein reference sequences downloaded from the UniProt database (https://www.uniprot.org/) and isoform sequences with 20,350 entries. Searches used carbamidomethylation as fixed modification and acetylation of the protein N-terminus, and oxidation of methionine as variable modifications. Default settings were used for other parameters.

### Clinical information about COVID-19 patients and control subjects

Clinical data including medical history, clinical manifestations, laboratory tests, radiological images, and antemortem findings of the patients with COVID-19 were obtained from medical records (Table 1). The control cohort included nine normal and age-matched adults, with causes of death not related to encephalitis, hypertension, myocardial infarction, atherosclerosis and cerebral ischemic stroke (Supplementary Table S1).

### Statistical analysis

Quantification values of identified proteins were normalized by taking the fraction of the total, which was multiplied by 10^6^ and log_2_ transformed. Pairwise comparisons to determine the proteins whose expression was significantly different between the COVID-19 patients and normal controls were performed by a moderated *t*-test using the R package limma (version 3.46.0).^31^ The differentially expressed proteins between COVID-19 (*n* = 7) and control (*n* = 8) brain samples were determined according to the Benjamin–Hochberg (BH) adjusted *p*-value and fold change values: BH adjusted *p*-value < 0.01 and log_2_ COVID-19/Control > 1 (upregulated), and *p*-value < 0.01 and log_2_ COVID-19/Control < −1 (downregulated). For other experiment data, the statistical analysis was done using SPSS (version 17 for windows, Inc.). The data from continuous variables are presented as means ± standard deviation (SD). Student *t*-test were performed for pairwise comparison. Pearson’s correlation coefficient test was used to assess the correlation of perivascular spaces with astrocyte activation involve different brain regions between COVID-19 patients and controls. A *p* < 0.05 was considered statistically significant.

## Supplementary information

Supplemental Fig.S3

Supplemental material

Supplemental Fig. S1

Supplemental Fig. S2

## Data Availability

All proteomics raw data have been deposited to the ProteomeXchange Consortium via the iProX partner1 repository with the dataset identifier PXD038748 (https://www.iprox.cn/page/PSV023.html;?url=16708572018119qUt).^48^
