# Supplementary figures and images for: COVID-19-associated monocytic encephalitis (CAME): histological and proteomic evidence from autopsy
Source: Signal Transduct Target Ther. 2023 Jan 6;8:24. doi: 10.1038/s41392-022-01291-6 (PMC9816522; doi:10.1038/s41392-022-01291-6)

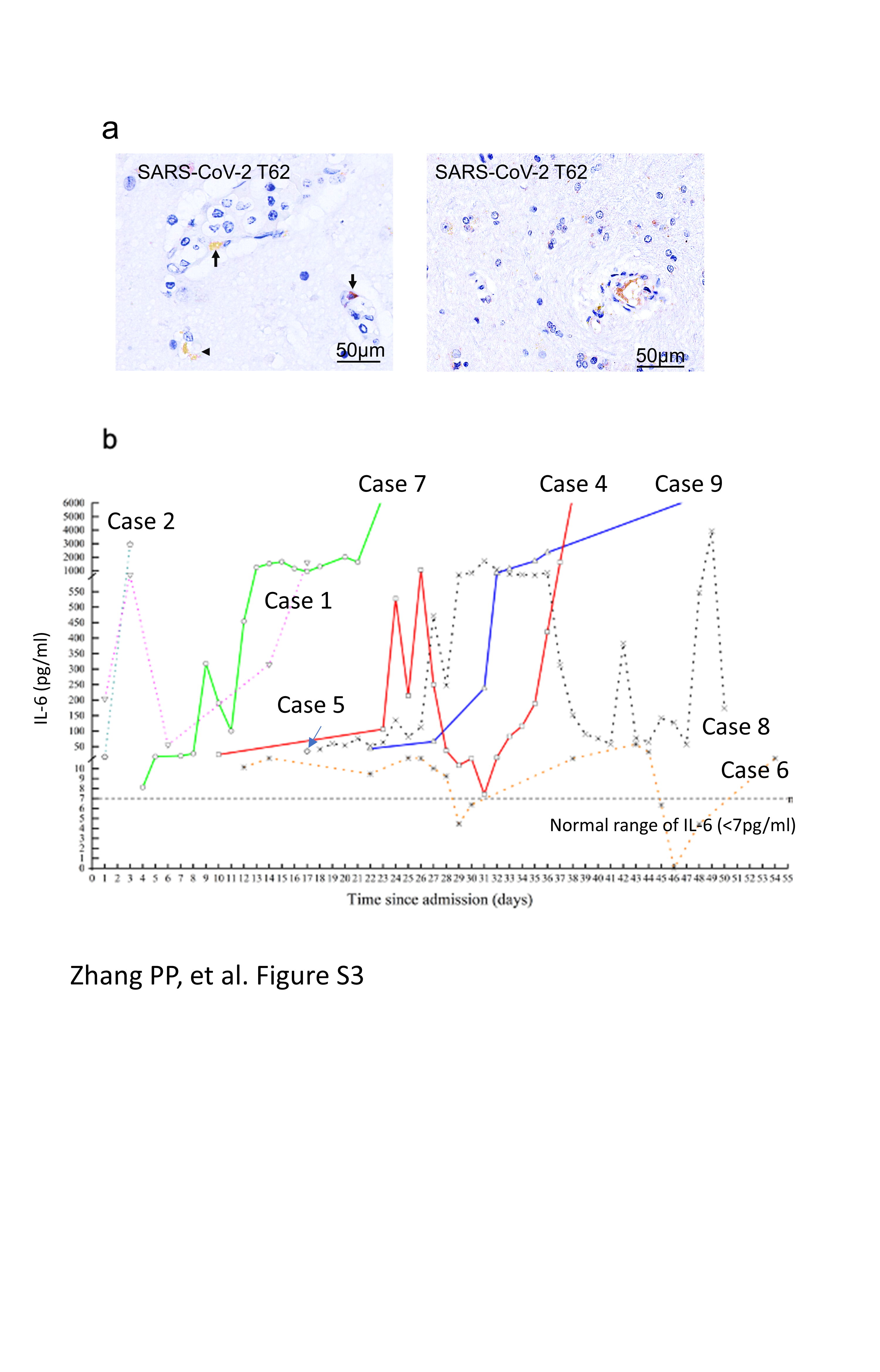

Supplement: Supplementary file 1 — Supplemental Fig.S3 [file 41392_2022_1291_MOESM1_ESM.tif]

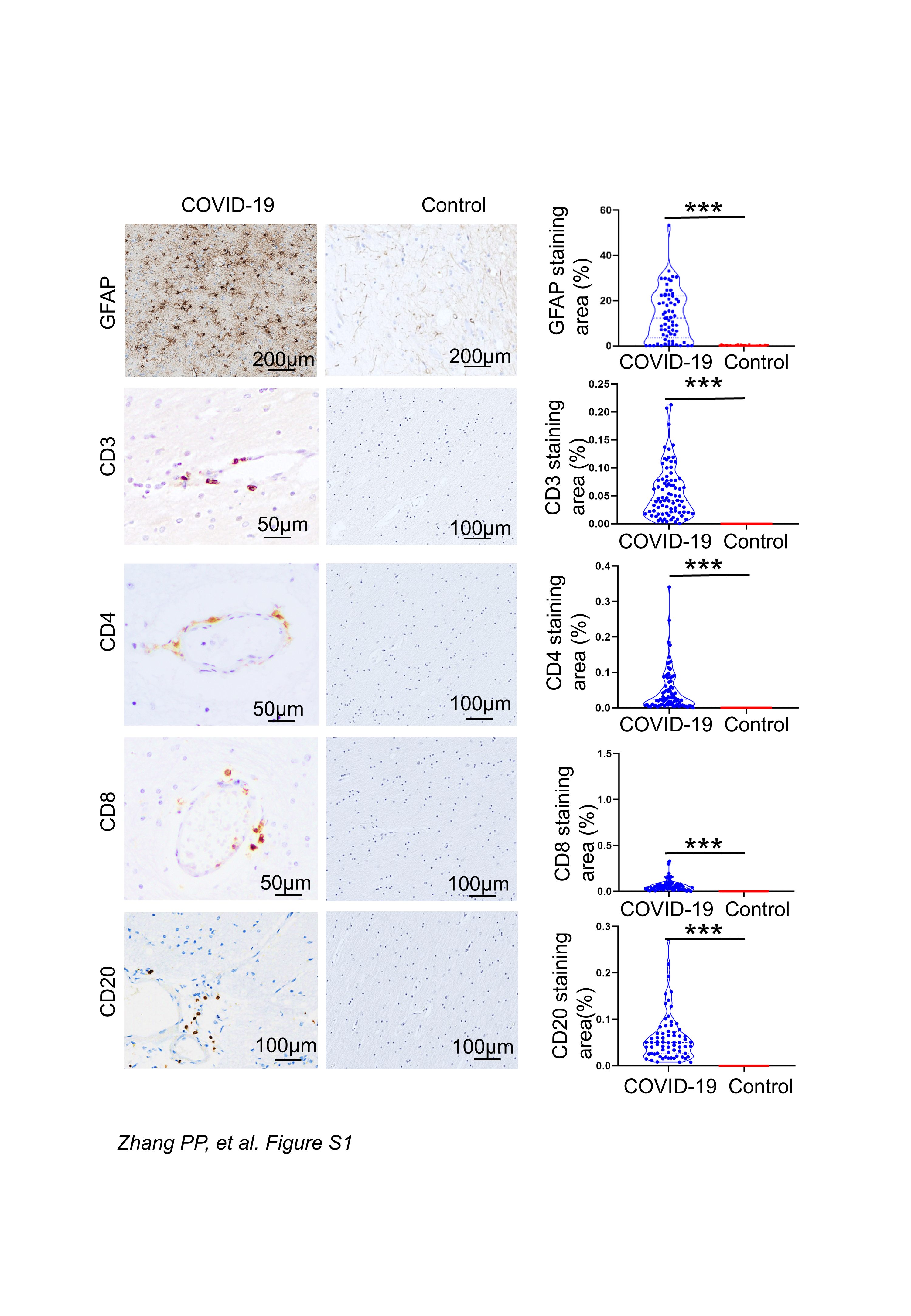

Supplement: Supplementary file 3 — Supplemental Fig. S1 [file 41392_2022_1291_MOESM3_ESM.tif]

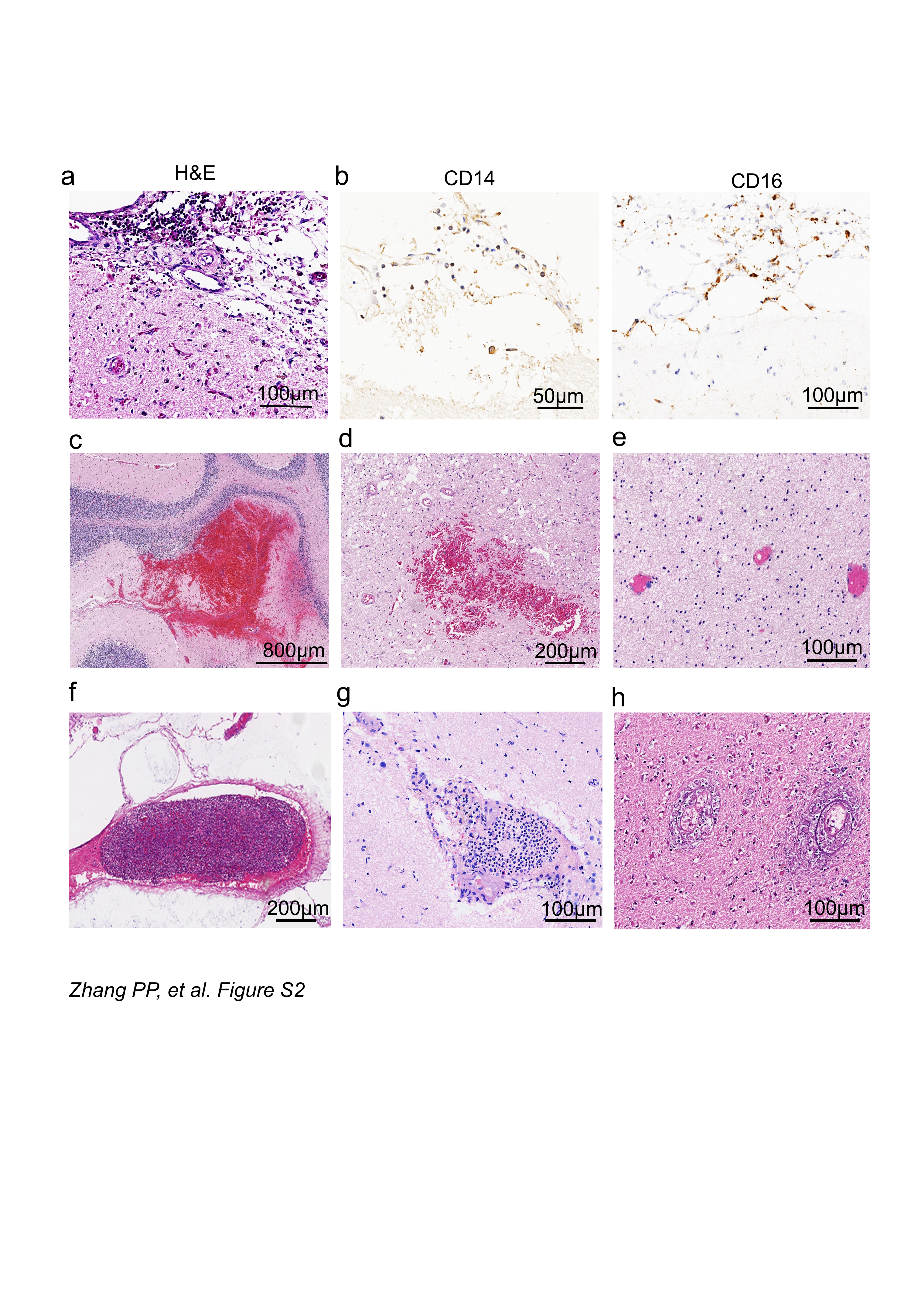

Supplement: Supplementary file 4 — Supplemental Fig. S2 [file 41392_2022_1291_MOESM4_ESM.tif]
