# Supplementary material for: COVID-19-associated monocytic encephalitis (CAME): histological and proteomic evidence from autopsy
Source: Signal Transduct Target Ther. 2023 Jan 6;8:24. doi: 10.1038/s41392-022-01291-6 (PMC9816522; doi:10.1038/s41392-022-01291-6)
Supplement: Supplementary file 2 — Supplemental material [file 41392_2022_1291_MOESM2_ESM.docx]

Supplementary Materials for

**COVID-19-associated monocytic encephalitis (CAME): histological and proteomic evidence from autopsy**

Pei-Pei Zhang, Zhi-Cheng He, Xiao-Hong Yao, Rui Tang, Jie Ma, Tao Luo, Chuhong Zhu, Tian-Ran Li, Xindong Liu, Dingyu Zhang, Shuyang Zhang, Yi-Fang Ping, Ling Leng, Xiu-Wu Bian

Correspondence to: [bianxiuwu@263.net](mailto:bianxiuwu@263.net)

**This PDF file includes:**

Materials and Methods

Figures. S1 to S3

Tables S1 to S4

**Materials and Methods**

**Quantitative analysis of immunohistochemical staining**

Digital section images of immunohistochemically slides were obtained using an Aperio Digital Pathology Slide Scanner (Aperio GT 450 DX) at 40x magnification (0.069 µm^2^ per raw image pixel). The obtained high-resolution digital images were managed using the Leica Image Scope software (version 12.4.0.5043). For further evaluation, at least five regions (1 mm x 1 mm) were randomly selected on each of the section images of CD3, CD4, CD8, CD20, CD14, CD16, IBA-1 and GFAP immunohistochemically stained slides. The selected microscopic image areas were decomposed into isolated individual stains (the red, green, blue OD color vectors) by color deconvolution algorithm, and were further converted into 8-bit HSV (Hue, Saturation, Value) images. Positively stained pixels were separated by thresholding the HSV images: H∈[90, 150], S∈[0, 255], V∈[69, 215]. Then, the interference regions caused by unspecific immunohistochemical staining were detected and removed from the selected image regions. Subsequently, total number of pixels in the IHC positive areas (*Pos*) and the total number of stained pixels in the selected image regions (*Total*) were extracted respectively. Staining was quantified by the metric: percentage of positive staining (% *Pos* = *Pos* / *Total* x 100%). The heatmap representing log2 mean % *Pos* value of each IHC marker after normalization was performed by R package (version 4.1.1). To measure the severity of perivascular spaces (PVS) enlargement, the area occupied by enlarged perivascular spaces was semi-automatically segmented and calculated.

**Proteomics data acquisition**

FFPE tissues were placed into EP tubes which were incubated at 60 ℃ metal bath for 10 min. Samples were then soaked in 1mL of xylene, vibrate for 10 min, centrifuge at 14000 rpm for 5 min. The supernatant was discarded. The process was repeated. The precipitate was then immersed in 1mL of 99% ethanol, 1mL of 96% ethanol and 1mL of 70% ethanol successively, vibrated for 5 min, centrifuged at 14000 rpm for 5 min for a total of 6 times. Finally, the samples were soaked in MS water and vibrated for 10 min, centrifuged at 14000 rpm for 5 min, and the supernatant was discarded. LB (100µL, 50% TFE,300mm Tris HCl) was added to each sample, and then treated with sonication (1 son, 1 s off, 20s, 30%). Then the test tube was incubated in 90 ℃ metal bath for 90 min. After heating, wait and the temperature of the metal bath decreased to below 60 ℃, and the test tube was carefully removed. Samples volume was replenished to 100µL with MS water and 30 µL sample were removed, final concentration of 5mm DTT was added and incubated for 20min at room temperature then alkylated in 25 mM iodoacetamide at room temperature for 20 min in darkness. 70 µL 10% TFE was added and 1 µg trypsin was incubated at 37 ℃ for 16 h. The second time, 1 µg trypsin was added and incubated at 37 ℃ for 4 h. The enzymatic hydrolysis was terminated with 50 µL 1% TFA. After centrifugation at 14000 rpm for 10 min, then the supernatants were transferred to a clean tube, followed by desalting through C18 cartridges (Beijing Qinglian Biotech Co., Ltd, China) and vacuum-dried by Speed Vac.

**Peptide pre-fractionation by high-pH HPLC**

Pool peptides were fractioned by using high-pH HPLC to reduce sample complexity. Briefly, peptides were dissolved in buffer A (2% acetonitrile (ACN), pH 9.5) and then loaded on an Xbridge C18 column (Waters, MA, 4.6 mm ×100 mm, 130A˚, 5μm) and eluted with a 70 min gradient from 0 to 95% buffer B (98% ACN, pH 9.5) at a flow rate of 0.7 mL/min. Aliquots were combined into 24 fractions before MS analysis.

**Mass spectrometry**

Samples were measured using an EASY-nLC 1200 (Thermo Fisher Scientific) coupled to a Q Exactive HF-X Orbitrap mass spectrometer (Thermo Fisher Scientific) via a nano-electrospray ion source (Thermo Fisher Scientific). Purified Peptides were re-dissolved in mobile phase A (20% ACN and 0.1% formic acid) and directly loaded onto an C18 nano-capillary analytical column (150µm ×150 mm, 100A˚ , 1.9µm, Beijing Qinglian Biotech Co.,Ltd). For the proteome profiling samples, peptides were separated on an analytical column over a 90-min gradient (buffer A: 0.1% formic acid and 80% H_2_O; buffer B: 0.1% formic acid and 20% acetonitrile (ACN) at a constant flow rate of 0.6 μL/min (0-15 min, 8 to 12% buffer B; 15-65 min, 12% to 30% buffer B; 65 to80 min, 30% to 40% buffer B; 81-90 min, 95% buffer B).

To acquire MS data, the data-independent acquisition (DIA) scan mode was used for single-shot samples, whereas the fractionated samples of the pool were acquired with a top 40 data-dependent acquisition (DDA) scan mode. Both acquisition schemes were combined with the same liquid chromatography gradient. The mass spectrometer was operated by the Xcalibur software (ThermoFisher). DDA scan settings on full MS level included an ion target value of 3 × 10^6^ charges in the 350–1,500 m/z range with a maximum injection time of 80 ms and a resolution of 120,000 at m/z 200. At the MS/MS level, the target value was 5 ×104 charges with a maximum injection time of 45 ms and a resolution of 15,000 at m/z 200. For MS/MS events only, precursor ions with 2-7 charges that were not on the 16s dynamic exclusion list were isolated in a 1.6 m/z window. Fragmentation was performed by higher-energy C-trap dissociation (HCD) with a normalized collision energy of 27 eV. DIA was performed with one full MS event followed by 42 MS/MS windows in one cycle. The full MS settings in cluded an ion target value of 3 × 10^6^ charges in the 350–1,500 m/z range with a maximum injection time of 50 ms and a resolution of 60,000 at m/z 200. DIA precursor windows ranged from 378 m/z (lower boundary of the first window) to 1345 m/z (upper boundary of the 42nd window). MS/MS settings included an ion target value of 1 × 10^6^ charges for the precursor window with an Xcalibur-automated maximum injection time and a resolution of 30,000 at m/z 200.

**Proteomic MS/MS data processing**

MS data of the fractionated pool (DDA MS data, 24 fractions) and single-shot samples (DIA MS data) were used to generate a DDA-library and direct-DIA-library, respectively, which were computationally merged into a hybrid library in the Spectronaut (version 14.9.201124.47784, Biognosys, Switzerland). Then, the raw DIA data were processed on Spectronaut using the default settings. All searches were performed against the human SP UniProt reference proteome of canonical and isoform sequences with 20,350 entries. Searches used carbamidomethylation as fixed modification and acetylation of the protein N-terminus, oxidation of methionines as variable modifications. Default settings were used for other parameters. Briefly, a trypsin/P proteolytic cleavage rule was used, permitting a maximum of two mis cleavages and a peptide length of 7–52 amino acids. Protein intensities were normalized using the “Local Normalization” algorithm in Spectronaut based on a local regression mode the retention time prediction type was set to dynamic iRT and correction factor for window. Mass calibration was set to local mass calibration. Decoy generation was set to Inverse. Interference correction on the MS2 level was enabled, removing fragments for quantification based on the presence of interfering signals but maintaining at least three fragments for quantification. The FDR was estimated with the mProphet approach and set to 1% at the peptide level.

**Bioinformatics analysis**

For proteins identified in COVID-19 brains and controls, the median normalization was used to reduce the biases between experiments, then the log2-transformed was performed and expression values were normally distributed. The proteins were quantified in seven COVID-19 brain samples and in seven control samples were remained for further statistical analysis. For analysis of differences in expression, an unpaired t-test, which implemented in the limma Package in R software (V3.46.0) was performed. The Benjamini-Hochberg (BH) procedure was implemented for multiple testing correction, and the adjusted *p* values were calculated to control the false discovery rate for each test. The differentially expressed proteins were defined as those with BH adjusted *p* value < 0.01 and a fold change of COVID-19/Control > 2 (significantly upregulated) or < 1/2 (significantly downregulated). The volcano plot was performed to illustrate changes in protein expression between COVID-19 brains and controls, showing that log2 fold change of COVID-19/Control (X-axis) with log10 BH adjusted *p* value (Y-axis).

Gene sets of the GO Biological Process (BP) ontology and canonical pathways were downloaded from the Molecular Signatures Database,^1^ and the identified proteins were annotated used the Python package scipy (version 1.2.0). The Principal Component Analysis (PCA) of the proteins whose values in each sample were valid was performed using the R package ape (version 5.4.1) The protein-protein interactions were retrieved from the STRING database, ^2^ and the network was built using Cytoscape (version 3.8.2). ^3^ We also used the circlize package (version 0.4.11) to circularly visualize the expression levels of inflammatory pathway related proteins in COVID-19 infected brain, lung and liver tissues.^4-6^

**References**

1. Subramanian, A., et al. Gene set enrichment analysis: a knowledge-based approach for interpreting genome-wide expression profiles. Proc. Natl. Acad. Sci. U. S. A. **102**, 15545-15550 (2005).

2. Szklarczyk, D., et al. The STRING database in 2017: quality-controlled protein-protein association networks, made broadly accessible. Nucleic Acids Res. **45**: D362-D368 (2017).

3. Shannon. P., et al. Cytoscape: a software environment for integrated models of biomolecular interaction networks. Genome Res. **13**, 2498-2504 (2003).

4. Gu, Z., et al. circlize Implements and enhances circular visualization in R. Bioinformatics **30**,2811-2812 (2014).

5. Leng, L., et al. Pathological features of COVID-19-associated lung injury: a preliminary proteomics report based on clinical samples. Signal Transduct. Target. Ther. **5**, 240 (2020).

6. Leng, L., et al. Pathological features of COVID-19-associated liver injury-a preliminary proteomics report based on clinical samples. Signal Transduct. Target. Ther. **6**, 9 (2021).

**Supplementary Fig. S1**

Representative images and quantitative analysis of immunohistochemical staining for activated astrocytes and infiltrating immune cells in the brain of COVID-19 patients and controls. Astrocytes are markedly positive for glial fibrillary acidic protein (GFAP), and perivascular infiltrating inflammatory cells are stained by anti-CD3, CD4, CD8, or CD20 in a representative temporal lobe of COVID-19 brains as compared with healthy controls. Statistics: *p<0.05, **p<0.01, ***p<0.001


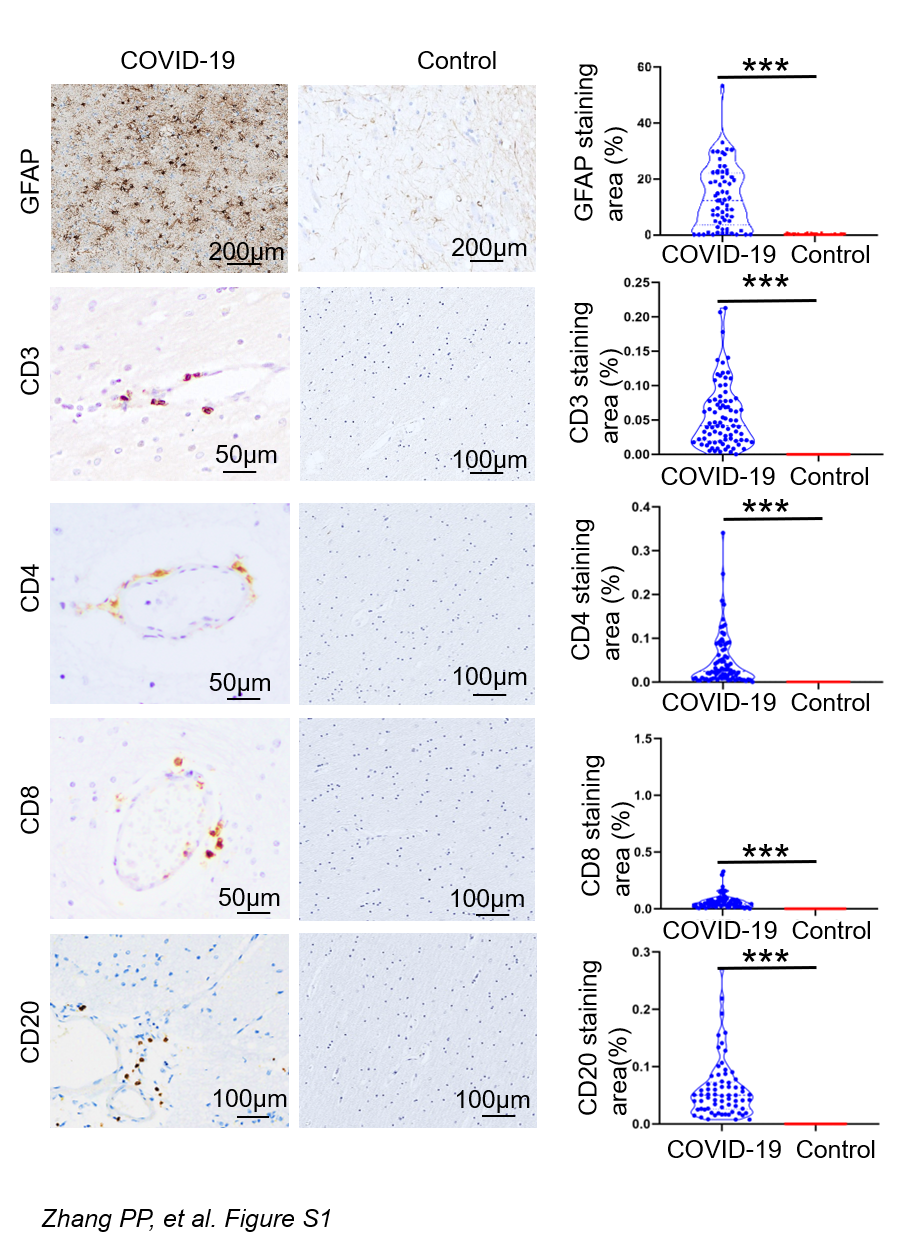


**Supplementary Fig. S2**

Pathological changes in COVID-19-associated monocytic encephalitis. **a** Meningoencephalitis in a COVID-19 brain. **b** Immunohistochemical staining of infiltrating immune cells in leptomeninges shown mainly as CD14+ and CD16+ monocytes. **c** and **d** Massive hemorrhage in cerebellar leptomeninges and parietal parenchyma. **e** and **f** Hyaline thrombosis within small vessels of brain parenchyma (**e**), and mixed thrombus in a meningeal vein (**f**). **g** Leukocyte accumulation within the lumen of small blood vessel in COVID-19 brain. **h** Necrotic cerebral vasculitis and perivasculitis.

**
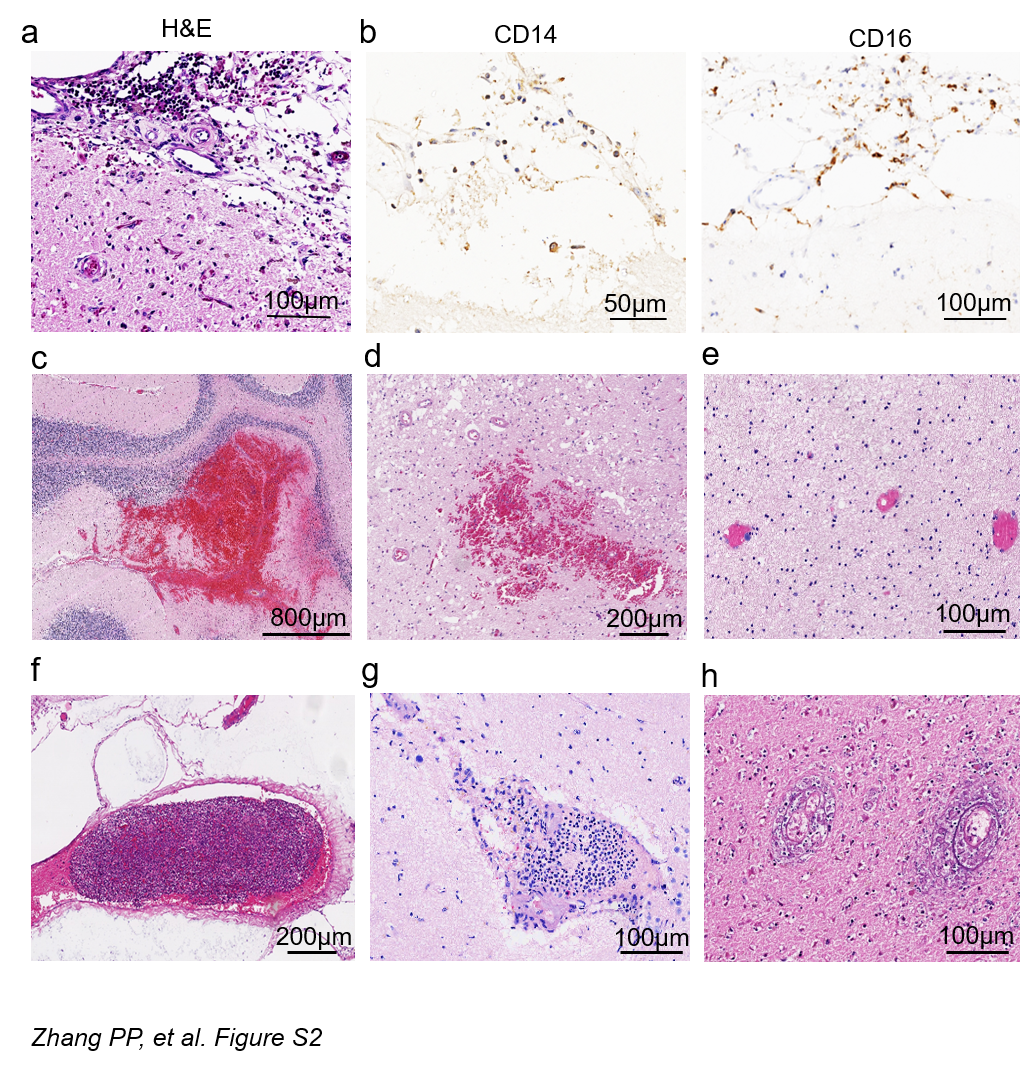
**

**Supplementary Fig. S3**

SARS-Cov-2 virus in microvascular endothelia and dynamic changes in interleukin-6 (IL-6) levels in peripheral blood of COVID-19 patients. **a** SARS-CoV-2 viral protein revealed in vascular endothelial cells of COVID-19 patient brain. **b** IL-6 level was significantly increased (*p* < 0.05) in peripheral blood of COVID-19 patients before death.


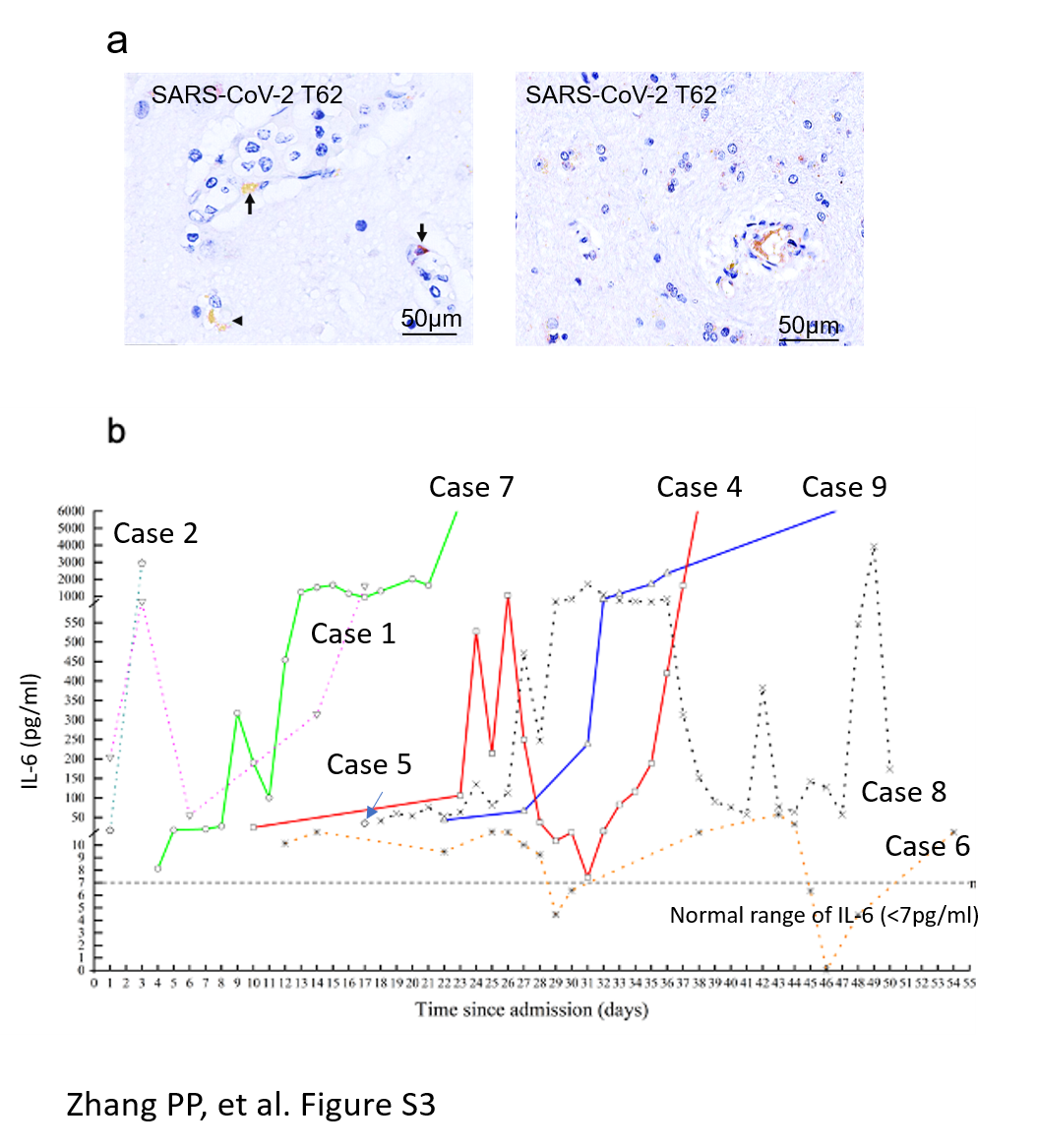


**Supplementary Table S1**

Clinical information of the control group

|  | case 1 | case 2 | case 3 | case 4 | case 5 | case 6 | case 7 | case 8 | case 9 |
| --- | --- | --- | --- | --- | --- | --- | --- | --- | --- |
| Sex | F | M | F | F | M | F | M | M | M |
| Age | 60 | 50 | 55 | 55 | 55 | 60 | 58 | 70 | 70 |
| Hypertension | No | No | No | No | No | No | No | No | Yes |
| Myocardial infarction | No | No | No | No | No | No | No | NA | No |
| Atherosclerosis | No | No | No | No | No | No | No | Yes | Yes |
| Cerebral ischemic stroke | No | No | No | No | No | No | No | NA | No |
| Pneumonia | No | No | No | No | No | No | No | No | No |
| Alzheimer disease | No | No | No | No | No | No | No | No | No |
| Parkinson’s disease | No | No | No | No | No | No | No | No | No |

F, female; M, male; NA, not available

**Supplementary Table S2**

The list of up-regulated and down-regulated proteins in COVID-19 brains compared to control brains

| Up-regulated proteins | logFC | t | P.Value | adj.P.Val |  | Down-regulated proteins | logFC | t | P.Value | adj.P.Val |
| --- | --- | --- | --- | --- | --- | --- | --- | --- | --- | --- |
| MT-ATP8 | 6.904401893 | 6.168083904 | 0.00009 | 0.001891895 |  | TOMM7 | -8.435783539 | -8.286167997 | 0.00001 | 0.000494664 |
| RAC2 | 5.286627005 | 8.816498190 | 0.00000 | 0.000262285 |  | EIF3M | -6.363091710 | -6.955400931 | 0.00006 | 0.001436589 |
| TSR2 | 5.261117124 | 9.977803590 | 0.00000 | 0.000301011 |  | PP2D1 | -5.593209940 | -7.78293972 | 0.00009 | 0.001824252 |
| ABCA2 | 4.968563948 | 6.796306406 | 0.00000 | 0.000301011 |  | FAM234B | -4.998940976 | -6.025368348 | 0.00045 | 0.005031204 |
| TUBA1C | 4.759230089 | 5.308094040 | 0.00023 | 0.003364973 |  | LUC7L3 | -4.641818325 | -9.392286391 | 0.00001 | 0.000585773 |
| GAP43 | 4.461984317 | 6.755353679 | 0.00001 | 0.00062177 |  | DOCK5 | -4.632873452 | -6.229081205 | 0.00022 | 0.003206347 |
| GOLGA2 | 4.457088667 | 4.457747846 | 0.00090 | 0.007580518 |  | MAP4K4 | -4.165597370 | -9.292637855 | 0.00001 | 0.000443315 |
| MARCKSL1 | 4.427761332 | 7.115545851 | 0.00000 | 0.000265773 |  | GRIN2A | -4.145139162 | -4.197902837 | 0.00099 | 0.008050434 |
| PLP1 | 4.354472833 | 6.414832447 | 0.00001 | 0.00046554 |  | HEXB | -4.003745018 | -5.765346499 | 0.00004 | 0.001312712 |
| TMSB10 | 4.159851088 | 8.736471184 | 0.00000 | 0.00015531 |  | COMMD10 | -3.933137156 | -6.867640508 | 0.00000 | 0.000338049 |
| SLC1A2 | 4.14679172 | 6.270729380 | 0.00001 | 0.000498622 |  | PDE4DIP | -3.932599155 | -5.500302495 | 0.00129 | 0.009572853 |
| PALM2 | 4.071642443 | 4.506973136 | 0.00105 | 0.008344646 |  | LRRC75A | -3.926802855 | -6.845321027 | 0.00004 | 0.00122648 |
| ARID2 | 3.941982453 | 7.170113099 | 0.00000 | 0.000236677 |  | FAM81A | -3.742869249 | -6.700254832 | 0.00013 | 0.002322258 |
| OCIAD1 | 3.756570934 | 5.698512196 | 0.00018 | 0.002767105 |  | GRSF1 | -3.699544342 | -5.870694024 | 0.00014 | 0.00245823 |
| LYN | 3.684273013 | 4.547538401 | 0.00078 | 0.006961461 |  | RPP30 | -3.688552192 | -7.153086722 | 0.00000 | 0.000262285 |
| PSAP | 3.667537968 | 6.794725310 | 0.00001 | 0.000606612 |  | IGHG4 | -3.680872225 | -6.336733395 | 0.00001 | 0.000622109 |
| DNAJC8 | 3.64249313 | 6.002689447 | 0.00018 | 0.002762003 |  | IGKV1-27 | -3.586227202 | -7.401291994 | 0.00012 | 0.002284735 |
| DAO | 3.552574255 | 5.709157925 | 0.00063 | 0.006141705 |  | NIPSNAP3B | -3.518995860 | -5.255480602 | 0.00047 | 0.005213594 |
| TMSB4X | 3.536250662 | 5.787081871 | 0.00003 | 0.00095932 |  | TMEM50A | -3.434770978 | -5.906073125 | 0.00002 | 0.000810474 |
| CIBAR2 | 3.505255456 | 4.110551773 | 0.00079 | 0.006985962 |  | THBS1 | -3.386105031 | -4.844366326 | 0.00030 | 0.003933388 |
| TPI1 | 3.45411229 | 7.726378151 | 0.00000 | 0.00015531 |  | SMC1A | -3.366320865 | -7.794125377 | 0.00000 | 0.00015531 |
| DNAJC5 | 3.303106837 | 4.387668429 | 0.00102 | 0.008214719 |  | ARMT1 | -3.348054987 | -4.514183954 | 0.00067 | 0.00635914 |
| BASP1 | 3.251689354 | 6.563075051 | 0.00000 | 0.000392585 |  | TAOK2 | -3.202445120 | -4.141811971 | 0.00084 | 0.007231176 |
| CEND1 | 3.182108916 | 4.407402463 | 0.00037 | 0.004472813 |  | RILPL1 | -3.179680724 | -4.308640243 | 0.00052 | 0.005489775 |
| TPM2 | 3.168871096 | 5.048975129 | 0.00046 | 0.005099088 |  | SCLY | -3.103243454 | -4.163383009 | 0.00092 | 0.007590407 |
| COX6C | 3.162728224 | 8.034910014 | 0.00000 | 0.00015531 |  | EDC4 | -3.047696645 | -5.256314225 | 0.00006 | 0.001468396 |
| HNRNPL | 3.153618564 | 6.450576224 | 0.00001 | 0.000450037 |  | TMPPE | -2.983877470 | -7.458689164 | 0.00000 | 0.000300345 |
| KRT4 | 3.115772003 | 6.618153829 | 0.00000 | 0.00036882 |  | TAF9 | -2.956364377 | -4.30653433 | 0.00096 | 0.007887777 |
| SLC25A4 | 2.951017277 | 4.319384247 | 0.00045 | 0.005031204 |  | TMEM201 | -2.953623293 | -5.808394291 | 0.00056 | 0.005774247 |
| RPLP2 | 2.941721209 | 8.311967904 | 0.00000 | 0.000180528 |  | CAVIN1 | -2.915393066 | -4.921456399 | 0.00026 | 0.003649778 |
| SNCA | 2.91973403 | 9.822411874 | 0.00000 | 2.47E-05 |  | NECAP2 | -2.900926169 | -4.967446593 | 0.00013 | 0.002379705 |
| VAPB | 2.911752457 | 5.631057009 | 0.00014 | 0.002449159 |  | ARHGEF4 | -2.890468799 | -7.352625919 | 0.00001 | 0.00062486 |
| MARCKS | 2.907712159 | 6.106180780 | 0.00001 | 0.00059347 |  | COMMD5 | -2.852987501 | -5.32833953 | 0.00030 | 0.00393525 |
| SVIP | 2.900272279 | 6.495848212 | 0.00001 | 0.000437843 |  | RFTN1 | -2.737723850 | -5.169634737 | 0.00007 | 0.00160026 |
| LGALS1 | 2.889684387 | 5.222896637 | 0.00007 | 0.001531994 |  | IGHV3-74 | -2.645785752 | -4.758559168 | 0.00024 | 0.003476507 |
| CD59 | 2.881181731 | 10.816598800 | 0.00000 | 8.73E-06 |  | RAB17 | -2.635105136 | -4.548918931 | 0.00027 | 0.003732871 |
| ALDH1B1 | 2.870857741 | 4.311753106 | 0.00080 | 0.007054232 |  | CDH20 | -2.565946964 | -4.621188515 | 0.00088 | 0.007457167 |
| MOBP | 2.86652446 | 4.457007529 | 0.00033 | 0.004159849 |  | NTMT1 | -2.565076696 | -4.710290626 | 0.00022 | 0.003336281 |
| CSRP1 | 2.8663404 | 5.126947246 | 0.00010 | 0.001934669 |  | TTC38 | -2.564276075 | -6.539301098 | 0.00001 | 0.000466683 |
| PPIAL4G | 2.824785787 | 4.902805200 | 0.00043 | 0.004925492 |  | PKP4 | -2.534996466 | -3.811530746 | 0.00136 | 0.009937033 |
| NECAB1 | 2.780835758 | 4.772858578 | 0.00017 | 0.002698591 |  | COMMD2 | -2.521937196 | -7.730731453 | 0.00000 | 0.000208507 |
| VIPR1 | 2.779361477 | 9.329733710 | 0.00000 | 3.95E-05 |  | MESD | -2.475279305 | -6.126644729 | 0.00001 | 0.000664393 |
| NDUFA7 | 2.777545596 | 4.285498059 | 0.00072 | 0.006630223 |  | LRRC7 | -2.462876472 | -6.307859492 | 0.00002 | 0.0007682 |
| TXNRD2 | 2.761828588 | 4.991572261 | 0.00038 | 0.004490519 |  | GCDH | -2.431687829 | -4.85537967 | 0.00014 | 0.002460345 |
| THY1 | 2.747790152 | 12.491927770 | 0.00000 | 1.89E-06 |  | SERPINC1 | -2.408358999 | -6.205899115 | 0.00001 | 0.000527499 |
| NEDD8 | 2.743778709 | 7.876104514 | 0.00000 | 0.000180528 |  | BRINP2 | -2.401175210 | -5.783100761 | 0.00003 | 0.001137551 |
| SKP1 | 2.739122992 | 6.386206276 | 0.00001 | 0.000466683 |  | CETN2 | -2.393890694 | -6.453716369 | 0.00001 | 0.000494664 |
| NKX2-8 | 2.734482478 | 6.085850541 | 0.00001 | 0.000606612 |  | IGKV3-15 | -2.377196404 | -4.585377164 | 0.00034 | 0.004210557 |
| NPY | 2.728998502 | 5.699759795 | 0.00003 | 0.001064521 |  | MCU | -2.349915895 | -5.577603853 | 0.00005 | 0.001368061 |
| ADGRG1 | 2.692641649 | 4.354309321 | 0.00089 | 0.007481801 |  | DHRS11 | -2.348156228 | -4.160198381 | 0.00125 | 0.00946503 |
| S100A16 | 2.691006432 | 6.715108044 | 0.00003 | 0.001031113 |  | CD38 | -2.347118806 | -4.694531931 | 0.00033 | 0.004109812 |
| FXYD6 | 2.61132604 | 4.520937078 | 0.00054 | 0.005627478 |  | C5orf51 | -2.329837275 | -6.625968337 | 0.00001 | 0.000443334 |
| HDGFL3 | 2.601189001 | 7.045331832 | 0.00001 | 0.000498622 |  | PAK5 | -2.294422224 | -5.420475497 | 0.00005 | 0.001390708 |
| SUCLG1 | 2.597823398 | 3.953720593 | 0.00123 | 0.00935473 |  | INPP5F | -2.283151056 | -5.22089925 | 0.00008 | 0.001696048 |
| GLTP | 2.595389373 | 5.413182461 | 0.00007 | 0.001562299 |  | BRINP1 | -2.279009331 | -4.876520498 | 0.00028 | 0.003814136 |
| ZYX | 2.594009061 | 4.351742544 | 0.00108 | 0.008493473 |  | ULK3 | -2.258759274 | -6.44178713 | 0.00001 | 0.000450037 |
| MANSC4 | 2.585184852 | 7.039975135 | 0.00001 | 0.000498622 |  | EXOSC6 | -2.249172924 | -5.103400554 | 0.00031 | 0.003984419 |
| RPLP1 | 2.577890494 | 4.969064793 | 0.00024 | 0.003475087 |  | PGS1 | -2.244564755 | -4.848156418 | 0.00014 | 0.002461304 |
| NPC2 | 2.550838351 | 6.040503179 | 0.00011 | 0.002147498 |  | C9 | -2.209013250 | -6.405651629 | 0.00001 | 0.000501643 |
| NAPG | 2.532009858 | 5.936757534 | 0.00002 | 0.000721989 |  | MRTO4 | -2.206916385 | -4.527145546 | 0.00029 | 0.003817244 |
| MT-ND2 | 2.531896016 | 5.537762599 | 0.00007 | 0.001562299 |  | SLC4A8 | -2.198157831 | -7.709716756 | 0.00000 | 0.00015531 |
| DNAJA4 | 2.525512361 | 7.451323167 | 0.00000 | 0.000180528 |  | MRI1 | -2.164894396 | -5.520463279 | 0.00004 | 0.001164421 |
| TMEM275 | 2.501949785 | 5.889155655 | 0.00005 | 0.001361833 |  | LUM | -2.145266984 | -5.242121884 | 0.00008 | 0.001649472 |
| TUBAL3 | 2.45331202 | 7.369794989 | 0.00000 | 0.000185918 |  | DNAJB11 | -2.134142904 | -6.122652275 | 0.00002 | 0.00077751 |
| COX4I1 | 2.433427301 | 7.490161469 | 0.00000 | 0.000180528 |  | VPS35L | -2.110766349 | -6.158203278 | 0.00002 | 0.0008622 |
| BANF1 | 2.429880269 | 4.868949865 | 0.00046 | 0.005110364 |  | INPPL1 | -2.088945692 | -5.508385037 | 0.00012 | 0.002303531 |
| DPYS | 2.418937737 | 7.218436127 | 0.00000 | 0.000225278 |  | EBNA1BP2 | -2.077640642 | -4.05151016 | 0.00089 | 0.007517464 |
| IDH2 | 2.415407828 | 5.292755675 | 0.00006 | 0.001436589 |  | KCTD21 | -2.073302172 | -5.928826953 | 0.00002 | 0.000790239 |
| MBP | 2.40979562 | 5.234982365 | 0.00006 | 0.001518407 |  | LRP1B | -2.048979075 | -4.11350572 | 0.00117 | 0.008952971 |
| NDUFB3 | 2.373561365 | 6.535070245 | 0.00002 | 0.000935808 |  | BTN2A1 | -2.030241533 | -5.362619789 | 0.00006 | 0.001468396 |
| CAMKV | 2.371641772 | 5.821116447 | 0.00002 | 0.000790114 |  | SNRPD2 | -2.001891305 | -4.632002647 | 0.00023 | 0.003364973 |
| H1-4 | 2.370312316 | 6.165844591 | 0.00001 | 0.000551668 |  | DNAJC12 | -1.995681519 | -4.761314333 | 0.00035 | 0.004278629 |
| ADPRS | 2.35506929 | 6.244353864 | 0.00001 | 0.000510078 |  | VPS41 | -1.989648229 | -5.044165572 | 0.00021 | 0.003163787 |
| CFL2 | 2.333321009 | 4.587783697 | 0.00025 | 0.003567699 |  | RASGRP2 | -1.981955122 | -4.86441395 | 0.00060 | 0.006052329 |
| RANBP1 | 2.318898997 | 4.550662639 | 0.00031 | 0.003983612 |  | GRIN1 | -1.966413215 | -5.544351583 | 0.00003 | 0.001137551 |
| SLC1A6 | 2.312742699 | 5.237280425 | 0.00008 | 0.001657337 |  | IGHV6-1 | -1.965239746 | -4.364982515 | 0.00073 | 0.006653227 |
| CD44 | 2.310239078 | 5.483590490 | 0.00004 | 0.001213126 |  | ZNRD2 | -1.957850954 | -4.123656665 | 0.00099 | 0.008039913 |
| CD81 | 2.304725788 | 5.203148688 | 0.00012 | 0.002311471 |  | SSB | -1.947559121 | -4.292757848 | 0.00054 | 0.005608378 |
| YTHDC2 | 2.302597735 | 5.907630625 | 0.00003 | 0.001164421 |  | ITGA3 | -1.943112405 | -4.323769699 | 0.00113 | 0.008764005 |
| KRT73 | 2.286954776 | 3.875911166 | 0.00118 | 0.009020221 |  | ADSL | -1.941274087 | -3.863015403 | 0.00134 | 0.009866571 |
| HSPA1L | 2.284919318 | 4.461707996 | 0.00113 | 0.008739261 |  | PIGS | -1.937566642 | -6.402986209 | 0.00001 | 0.000466683 |
| SYP | 2.282592965 | 5.820723711 | 0.00002 | 0.000790114 |  | SYNJ2BP | -1.916711961 | -4.458166697 | 0.00051 | 0.005453681 |
| FHL1 | 2.272941202 | 6.637203162 | 0.00001 | 0.000697867 |  | SLC6A7 | -1.916433724 | -4.508839229 | 0.00034 | 0.004210557 |
| SYNPO | 2.239677109 | 4.673389857 | 0.00021 | 0.003163787 |  | DCPS | -1.913737190 | -4.509113935 | 0.00034 | 0.004210557 |
| DYNC1LI2 | 2.237428014 | 7.777035241 | 0.00000 | 0.00015531 |  | PSMD5 | -1.908770304 | -4.232957738 | 0.00054 | 0.005627478 |
| MYG1 | 2.22952389 | 4.909018994 | 0.00013 | 0.002322258 |  | ADAM11 | -1.908608042 | -5.827919658 | 0.00002 | 0.000790114 |
| ATP5PF | 2.22927246 | 5.518007247 | 0.00004 | 0.001164421 |  | HECW2 | -1.895607401 | -4.490616785 | 0.00031 | 0.003961486 |
| OAS3 | 2.226220653 | 6.955571741 | 0.00000 | 0.000262285 |  | PLA2G4C | -1.878137193 | -4.084826156 | 0.00075 | 0.006786804 |
| NDUFA3 | 2.208738201 | 4.423676185 | 0.00065 | 0.006307518 |  | MFN1 | -1.870971211 | -4.183291379 | 0.00060 | 0.006052329 |
| METTL7A | 2.198538389 | 6.186772195 | 0.00001 | 0.00062177 |  | CHCHD6 | -1.857145797 | -7.478334358 | 0.00000 | 0.000180528 |
| GLOD4 | 2.195736828 | 8.291677064 | 0.00000 | 0.000127267 |  | LRG1 | -1.841897035 | -4.612467622 | 0.00038 | 0.004535789 |
| REEP2 | 2.194347107 | 4.295768849 | 0.00061 | 0.006079383 |  | GNS | -1.840709359 | -4.250213028 | 0.00107 | 0.0084262 |
| ATP6V1G2 | 2.174857198 | 5.161485752 | 0.00009 | 0.001848396 |  | NRP2 | -1.840483702 | -5.673251816 | 0.00005 | 0.001390708 |
| ATP12A | 2.166227282 | 4.856108807 | 0.00014 | 0.002460345 |  | TESC | -1.817088199 | -4.272867219 | 0.00102 | 0.008239525 |
| PGK1 | 2.16391651 | 6.353738226 | 0.00001 | 0.000488681 |  | MKLN1 | -1.802121515 | -4.818751983 | 0.00021 | 0.0032017 |
| SLC25A6 | 2.156663993 | 6.634248191 | 0.00000 | 0.00036882 |  | RTN4R | -1.800978065 | -4.134425526 | 0.00112 | 0.008727936 |
| GNG2 | 2.146530441 | 5.869867999 | 0.00002 | 0.000768083 |  | EPHX2 | -1.798235506 | -5.487822644 | 0.00004 | 0.001212149 |
| ITGAE | 2.119793383 | 5.630189675 | 0.00004 | 0.001310669 |  | SLC30A3 | -1.789851759 | -6.849244831 | 0.00000 | 0.000293953 |
| S100A1 | 2.100467111 | 4.926184559 | 0.00014 | 0.002461304 |  | NUDCD2 | -1.782299656 | -5.443704319 | 0.00004 | 0.001279509 |
| SGTB | 2.073982084 | 4.121181249 | 0.00135 | 0.009909805 |  | EVI5L | -1.776937182 | -4.747219087 | 0.00072 | 0.006630223 |
| HMGB1 | 2.068042813 | 5.680579799 | 0.00007 | 0.001567437 |  | KCNAB1 | -1.776066383 | -4.400548683 | 0.00049 | 0.005369094 |
| SNCG | 2.060472808 | 5.316462398 | 0.00005 | 0.001396297 |  | TRAPPC1 | -1.766227570 | -5.994142008 | 0.00002 | 0.000768083 |
| SF3B1 | 2.059540478 | 8.118367660 | 0.00000 | 0.000127267 |  | REEP6 | -1.761088780 | -5.221031611 | 0.00012 | 0.002284735 |
| CYB5B | 2.059262825 | 5.350327776 | 0.00005 | 0.00137033 |  | CCDC47 | -1.753034890 | -4.283261275 | 0.00055 | 0.005667321 |
| MYL6 | 2.059030763 | 7.512184168 | 0.00000 | 0.000180528 |  | SRP68 | -1.751145547 | -4.626424306 | 0.00031 | 0.003983612 |
| AOAH | 2.058380928 | 4.022845083 | 0.00107 | 0.0084262 |  | NECTIN1 | -1.749869628 | -6.226084796 | 0.00001 | 0.000521258 |
| PEBP1 | 2.056503809 | 8.132908898 | 0.00000 | 0.000127267 |  | PSMB5 | -1.748357683 | -6.031636087 | 0.00001 | 0.00062486 |
| DYNLL1 | 2.054580298 | 8.470344748 | 0.00000 | 0.000126443 |  | LIN7B | -1.731572850 | -4.851990698 | 0.00014 | 0.002460345 |
| REEP5 | 2.051495712 | 4.397416441 | 0.00043 | 0.004913209 |  | RAB2A | -1.725627334 | -5.349119841 | 0.00005 | 0.00137033 |
| PREPL | 2.046415579 | 4.033047496 | 0.00136 | 0.009937033 |  | CRTAC1 | -1.712832014 | -7.070471869 | 0.00000 | 0.000246471 |
| PSEN1 | 2.039441894 | 4.550448539 | 0.00027 | 0.003732871 |  | KSR1 | -1.706682472 | -5.122708436 | 0.00010 | 0.001934831 |
| CSTB | 2.038813425 | 5.647801228 | 0.00003 | 0.000986812 |  | PAAF1 | -1.679620971 | -5.17330084 | 0.00011 | 0.002095007 |
| CADM3 | 2.03538477 | 5.203304965 | 0.00007 | 0.001562299 |  | PCYOX1L | -1.677806503 | -4.957683326 | 0.00011 | 0.002191699 |
| EIF5A | 2.030226273 | 6.867241230 | 0.00000 | 0.000291861 |  | ADGRL3 | -1.672801992 | -4.405251698 | 0.00049 | 0.005335991 |
| HNRNPA3 | 2.024723424 | 5.813261916 | 0.00002 | 0.000790239 |  | RABGGTA | -1.667020764 | -4.764533128 | 0.00017 | 0.002725843 |
| NUTF2 | 2.010226258 | 5.350022566 | 0.00005 | 0.00137033 |  | STX8 | -1.660642439 | -6.308935522 | 0.00001 | 0.000494664 |
| ENO1 | 2.007488098 | 4.344425800 | 0.00042 | 0.004864929 |  | RPS16 | -1.645476341 | -3.994969037 | 0.00091 | 0.007581769 |
| HNRNPD | 1.998605796 | 8.444855745 | 0.00000 | 0.000127267 |  | ARFIP2 | -1.644685536 | -6.183059761 | 0.00001 | 0.000544303 |
| PGAM2 | 1.99198169 | 4.511172852 | 0.00083 | 0.00717953 |  | RAB4B | -1.642765956 | -6.96702534 | 0.00000 | 0.000262285 |
| ERH | 1.977487704 | 4.224835486 | 0.00055 | 0.005677292 |  | MCTS1 | -1.640227763 | -4.585609391 | 0.00025 | 0.003572762 |
| AGFG1 | 1.976014254 | 5.410318688 | 0.00014 | 0.002461304 |  | TMEM132A | -1.630327068 | -4.681406308 | 0.00021 | 0.003132511 |
| PFDN1 | 1.968051442 | 5.271267894 | 0.00006 | 0.001468396 |  | EIF2B5 | -1.621655884 | -6.455243096 | 0.00001 | 0.000494664 |
| SYNGR1 | 1.960763574 | 5.672314170 | 0.00003 | 0.00095932 |  | LNPEP | -1.617069009 | -5.328367329 | 0.00005 | 0.001390708 |
| CNOT2 | 1.960076372 | 5.770009240 | 0.00004 | 0.001310669 |  | TBC1D13 | -1.616453623 | -4.610075278 | 0.00028 | 0.003749337 |
| GLUD1 | 1.953103045 | 5.194172082 | 0.00007 | 0.001567437 |  | VWA5A | -1.603052493 | -3.991617426 | 0.00092 | 0.007590407 |
| AK1 | 1.951041054 | 7.100519860 | 0.00000 | 0.000246471 |  | CISD2 | -1.594277488 | -6.702196345 | 0.00000 | 0.000338049 |
| SNCB | 1.944425033 | 5.520875756 | 0.00004 | 0.001164421 |  | RPL35A | -1.590347601 | -6.76364449 | 0.00000 | 0.000312549 |
| GSPT1 | 1.937253113 | 4.287613123 | 0.00072 | 0.006630223 |  | NUDT16 | -1.581448630 | -5.422756849 | 0.00004 | 0.001308072 |
| PGAM1 | 1.933448295 | 3.953516021 | 0.00099 | 0.008050434 |  | CLSTN1 | -1.579857906 | -4.174243525 | 0.00061 | 0.006090282 |
| MICOS10 | 1.907086415 | 5.376321175 | 0.00005 | 0.001344286 |  | CPNE5 | -1.574383530 | -5.893992171 | 0.00002 | 0.000766261 |
| RPL29 | 1.904137791 | 5.429103259 | 0.00007 | 0.001531994 |  | CDH6 | -1.572605887 | -4.555712346 | 0.00077 | 0.006899608 |
| RPS20 | 1.886111571 | 4.296538441 | 0.00053 | 0.005590722 |  | TMEM9B | -1.571328797 | -5.759539734 | 0.00003 | 0.000986812 |
| SYNPR | 1.885366621 | 5.762988573 | 0.00006 | 0.001468396 |  | GOT2 | -1.568606195 | -4.903520218 | 0.00013 | 0.002322258 |
| MPC2 | 1.882607972 | 6.016498950 | 0.00003 | 0.001031113 |  | GRM3 | -1.567347981 | -4.974358008 | 0.00011 | 0.002144101 |
| ATP2B3 | 1.881014567 | 3.861845864 | 0.00134 | 0.009874385 |  | LZIC | -1.553554564 | -4.170464096 | 0.00079 | 0.006985962 |
| NDRG4 | 1.874555599 | 4.543642816 | 0.00028 | 0.003749337 |  | SELENOM | -1.550859969 | -4.948753782 | 0.00016 | 0.00268823 |
| MACF1 | 1.873140853 | 5.311983723 | 0.00005 | 0.001400977 |  | MT-ND5 | -1.549584849 | -5.176725145 | 0.00007 | 0.001586723 |
| RAB3C | 1.869474808 | 4.134834980 | 0.00075 | 0.006800089 |  | PRDX4 | -1.544700689 | -4.917213805 | 0.00012 | 0.002311471 |
| EEF1D | 1.858108462 | 4.907794622 | 0.00013 | 0.002322258 |  | KIAA1109 | -1.542023549 | -3.909035265 | 0.00135 | 0.009909805 |
| TUBB1 | 1.844388445 | 4.025113416 | 0.00085 | 0.007334032 |  | ASRGL1 | -1.537473306 | -4.679352228 | 0.00021 | 0.003135342 |
| TIMM13 | 1.820757195 | 4.265065284 | 0.00125 | 0.00946503 |  | ARHGEF12 | -1.536787082 | -6.162715986 | 0.00001 | 0.000551668 |
| ACAT1 | 1.815713345 | 4.780846495 | 0.00017 | 0.00268823 |  | RRAGC | -1.534304376 | -3.92550045 | 0.00117 | 0.008958363 |
| SHTN1 | 1.812684355 | 6.258393006 | 0.00002 | 0.000790114 |  | ATP5F1E | -1.533110676 | -4.579858703 | 0.00026 | 0.003605529 |
| PITPNA | 1.807751059 | 7.083806221 | 0.00000 | 0.000246471 |  | ZYG11B | -1.530311388 | -3.995213052 | 0.00091 | 0.007581769 |
| TARDBP | 1.798458398 | 4.518639822 | 0.00039 | 0.00457251 |  | TM9SF4 | -1.529668170 | -5.009280245 | 0.00012 | 0.002288937 |
| GLUL | 1.790364326 | 4.761327082 | 0.00017 | 0.002725843 |  | GFRA2 | -1.527537062 | -5.430794067 | 0.00005 | 0.001390708 |
| DNAJA1 | 1.788408321 | 6.017152844 | 0.00002 | 0.000766261 |  | NUP210 | -1.519065353 | -4.639976363 | 0.00036 | 0.004385744 |
| ARHGAP1 | 1.788295492 | 7.427099010 | 0.00000 | 0.000180528 |  | UPF1 | -1.519038130 | -4.806047664 | 0.00016 | 0.002621476 |
| ARHGAP44 | 1.781001024 | 4.108911217 | 0.00089 | 0.007517464 |  | EGFR | -1.510081922 | -4.327701966 | 0.00057 | 0.005858074 |
| VAPA | 1.772452428 | 5.570377705 | 0.00003 | 0.001095537 |  | SPON1 | -1.495527394 | -4.029638028 | 0.00084 | 0.007276299 |
| TUBB8 | 1.772145598 | 4.786799220 | 0.00016 | 0.002678496 |  | H4C1 | -1.495103007 | -6.325798225 | 0.00001 | 0.000494664 |
| CDC42 | 1.771284049 | 6.213496950 | 0.00001 | 0.000526893 |  | IQGAP1 | -1.492345025 | -4.803976657 | 0.00016 | 0.002621476 |
| CHD6 | 1.770819988 | 4.572900198 | 0.00041 | 0.00474005 |  | TRAPPC6B | -1.490105471 | -4.833646424 | 0.00025 | 0.003564552 |
| STMN2 | 1.769443418 | 6.617437305 | 0.00000 | 0.00036882 |  | CACNG3 | -1.484446438 | -4.884640566 | 0.00013 | 0.002380225 |
| UTP20 | 1.759352785 | 5.257265279 | 0.00006 | 0.001468396 |  | SERPINI1 | -1.481635203 | -4.142868746 | 0.00066 | 0.00633486 |
| NCAM1 | 1.758400587 | 5.597118317 | 0.00003 | 0.001053888 |  | PMM1 | -1.480158483 | -4.434993629 | 0.00040 | 0.004650534 |
| LTA4H | 1.754876005 | 3.965168514 | 0.00097 | 0.007921523 |  | MBLAC2 | -1.477730083 | -4.257275244 | 0.00051 | 0.005453681 |
| S100B | 1.745321553 | 5.200004248 | 0.00007 | 0.00156485 |  | PPP1R12C | -1.472602383 | -4.151327409 | 0.00109 | 0.008513839 |
| MAP4 | 1.735156383 | 7.052586047 | 0.00000 | 0.000246549 |  | WASHC2A | -1.471737904 | -4.157516311 | 0.00107 | 0.0084451 |
| TAF15 | 1.730369847 | 5.386384833 | 0.00006 | 0.001436589 |  | CCT5 | -1.470921788 | -5.959329055 | 0.00001 | 0.000697867 |
| AMPH | 1.722291881 | 6.604784939 | 0.00000 | 0.000369981 |  | ADAM23 | -1.456446961 | -5.658104979 | 0.00003 | 0.000977778 |
| RPS27L | 1.718281249 | 5.003515914 | 0.00015 | 0.002509312 |  | BCAN | -1.455712392 | -5.228902928 | 0.00006 | 0.001529415 |
| FAM98A | 1.709694161 | 4.953632419 | 0.00025 | 0.00353755 |  | MRPS34 | -1.448982439 | -4.568204214 | 0.00026 | 0.003649778 |
| NKTR | 1.709032104 | 4.894012015 | 0.00034 | 0.00422569 |  | KNDC1 | -1.443724837 | -4.464566977 | 0.00051 | 0.005442069 |
| TOMM70 | 1.703258095 | 5.063965392 | 0.00009 | 0.001875214 |  | WDR11 | -1.443215435 | -4.217444094 | 0.00096 | 0.007853212 |
| SUCLA2 | 1.701509755 | 4.088086136 | 0.00074 | 0.006766937 |  | CARHSP1 | -1.439302297 | -4.513026996 | 0.00039 | 0.004601137 |
| MAP2K2 | 1.695123888 | 3.845862472 | 0.00126 | 0.009483319 |  | ACOT1 | -1.431132290 | -4.271010124 | 0.00050 | 0.005403621 |
| HNRNPK | 1.692560316 | 4.555439044 | 0.00027 | 0.003715831 |  | LYZ | -1.430372788 | -4.156151971 | 0.00064 | 0.006240516 |
| ALDH8A1 | 1.688527826 | 4.181611516 | 0.00068 | 0.006455591 |  | ITIH5 | -1.429574581 | -4.100583731 | 0.00091 | 0.007581769 |
| OPTN | 1.679986251 | 5.680695270 | 0.00005 | 0.001390708 |  | MON2 | -1.426884237 | -5.053984615 | 0.00009 | 0.001891895 |
| APOD | 1.67357534 | 5.038064583 | 0.00010 | 0.001934669 |  | PSMA4 | -1.422040129 | -4.101445005 | 0.00072 | 0.006630223 |
| CANX | 1.671693569 | 5.873669351 | 0.00002 | 0.000768083 |  | EEA1 | -1.420748752 | -5.920723299 | 0.00002 | 0.000737459 |
| DPYSL4 | 1.659632503 | 4.778113147 | 0.00017 | 0.00268823 |  | TRIO | -1.418636308 | -4.00248796 | 0.00089 | 0.007517464 |
| TMEM205 | 1.656378693 | 4.131722941 | 0.00097 | 0.007927704 |  | ADCY1 | -1.417436257 | -4.852433221 | 0.00014 | 0.002460345 |
| MRPS36 | 1.65470918 | 4.983439202 | 0.00013 | 0.002322258 |  | HUWE1 | -1.404248410 | -4.98662007 | 0.00011 | 0.002098578 |
| GLRX3 | 1.651869039 | 4.578051929 | 0.00041 | 0.004718106 |  | WASF3 | -1.402508902 | -5.383173222 | 0.00005 | 0.001336234 |
| KIF5B | 1.644322579 | 4.997711447 | 0.00012 | 0.002311471 |  | LDAH | -1.400990632 | -4.573772829 | 0.00030 | 0.003922208 |
| KIAA0513 | 1.638390999 | 3.942422465 | 0.00102 | 0.008217581 |  | OPHN1 | -1.399755526 | -4.59912056 | 0.00024 | 0.003527817 |
| ERP29 | 1.635107617 | 5.263427614 | 0.00006 | 0.001468396 |  | GOLGA7B | -1.397211134 | -4.736493566 | 0.00021 | 0.0032017 |
| RTN1 | 1.628387661 | 5.090926057 | 0.00009 | 0.001814683 |  | LYRM4 | -1.396518748 | -4.457238645 | 0.00033 | 0.004159849 |
| CRYAB | 1.599258189 | 3.932053306 | 0.00104 | 0.008328954 |  | VPS26C | -1.394828347 | -4.836824538 | 0.00015 | 0.002501422 |
| ATP5IF1 | 1.597542698 | 5.487564441 | 0.00004 | 0.001212149 |  | PLCL2 | -1.381490912 | -4.122423097 | 0.00069 | 0.00649637 |
| ACADM | 1.5952478 | 4.033573608 | 0.00093 | 0.007661005 |  | CAMSAP2 | -1.379708018 | -4.392420539 | 0.00050 | 0.005417606 |
| RAP1B | 1.594131893 | 5.882464780 | 0.00002 | 0.000768083 |  | CACNA2D3 | -1.377457883 | -4.837800073 | 0.00017 | 0.002725843 |
| PRPSAP2 | 1.586995324 | 4.429511485 | 0.00035 | 0.004315603 |  | AGO1 | -1.377121905 | -4.732507931 | 0.00030 | 0.003935624 |
| RBBP4 | 1.586143922 | 4.829361355 | 0.00031 | 0.003961486 |  | FSCN1 | -1.373452782 | -6.271012399 | 0.00001 | 0.000498622 |
| DECR1 | 1.585336369 | 4.293326616 | 0.00047 | 0.005241465 |  | SCFD1 | -1.371002989 | -5.804706093 | 0.00002 | 0.000790239 |
| RPS15 | 1.581391726 | 3.939402949 | 0.00103 | 0.008241504 |  | MFSD4A | -1.367017397 | -4.3853895 | 0.00039 | 0.00457251 |
| CAP1 | 1.579357703 | 4.518864258 | 0.00029 | 0.003861438 |  | PSMB6 | -1.363194876 | -4.514779041 | 0.00029 | 0.003883793 |
| CACNB3 | 1.576000135 | 4.628173709 | 0.00023 | 0.003369931 |  | MKRN2 | -1.358568865 | -4.042702145 | 0.00091 | 0.007585956 |
| MAP7D1 | 1.57525775 | 4.321530805 | 0.00045 | 0.005030023 |  | HARS1 | -1.356977002 | -4.546280599 | 0.00027 | 0.003742395 |
| TOMM22 | 1.574369733 | 4.035909644 | 0.00092 | 0.007652127 |  | RPL15 | -1.353264758 | -5.280186833 | 0.00006 | 0.001453611 |
| HEBP1 | 1.567532557 | 7.129263501 | 0.00000 | 0.000245693 |  | MTM1 | -1.349226977 | -5.466887853 | 0.00004 | 0.001228515 |
| YWHAG | 1.563893587 | 5.026623251 | 0.00010 | 0.001963789 |  | COPS7A | -1.343637217 | -5.260887454 | 0.00006 | 0.001468396 |
| TTN | 1.562650932 | 3.895883849 | 0.00113 | 0.008742059 |  | GPR37L1 | -1.342266570 | -5.747739208 | 0.00002 | 0.000855033 |
| CFL1 | 1.544562652 | 5.747091672 | 0.00002 | 0.000855033 |  | ERP44 | -1.340440175 | -5.382975577 | 0.00005 | 0.001336234 |
| COX6B1 | 1.542465925 | 4.181784131 | 0.00060 | 0.006052329 |  | LRFN3 | -1.326427962 | -4.326022899 | 0.00057 | 0.005864296 |
| CISD1 | 1.540924357 | 5.802127709 | 0.00002 | 0.000790239 |  | CREG1 | -1.325446227 | -4.998311023 | 0.00010 | 0.002075359 |
| RTTN | 1.53864149 | 5.576118981 | 0.00004 | 0.001228515 |  | ATP2C1 | -1.323132006 | -3.848359274 | 0.00125 | 0.00946503 |
| ACOT13 | 1.53689968 | 4.903114830 | 0.00013 | 0.002322258 |  | SUOX | -1.321118355 | -4.498830566 | 0.00030 | 0.003936099 |
| ACTA1 | 1.513285939 | 4.298252041 | 0.00047 | 0.005213594 |  | TRIM46 | -1.306606406 | -4.332784035 | 0.00044 | 0.004934193 |
| STX4 | 1.510364921 | 4.856062709 | 0.00017 | 0.00268823 |  | AP2M1 | -1.304155019 | -4.614339407 | 0.00024 | 0.003453217 |
| TIMM22 | 1.49794814 | 5.573637521 | 0.00006 | 0.001518407 |  | RPS14 | -1.294035018 | -4.104720261 | 0.00072 | 0.006630223 |
| CZIB | 1.494023168 | 4.453353002 | 0.00062 | 0.006090282 |  | NOMO3 | -1.291256772 | -4.367771137 | 0.00040 | 0.004683283 |
| UXT | 1.493580394 | 4.467422246 | 0.00043 | 0.004913209 |  | VPS51 | -1.285461455 | -5.179930945 | 0.00007 | 0.001586723 |
| RBMX | 1.488015141 | 4.171954051 | 0.00062 | 0.006095837 |  | DENND4B | -1.281523058 | -4.379637987 | 0.00039 | 0.004601137 |
| RPS27A | 1.481786466 | 4.993620786 | 0.00011 | 0.002086522 |  | CDK5RAP3 | -1.280931275 | -4.073281093 | 0.00077 | 0.006899608 |
| DBI | 1.477269785 | 5.604416003 | 0.00003 | 0.001046713 |  | TM7SF2 | -1.280315186 | -4.679925272 | 0.00033 | 0.004178135 |
| PSD3 | 1.471733824 | 3.881125033 | 0.00117 | 0.008952971 |  | TIGAR | -1.275625376 | -4.08674234 | 0.00083 | 0.007198971 |
| ESD | 1.460576059 | 4.589949218 | 0.00025 | 0.003564552 |  | ANXA4 | -1.272693864 | -4.775011403 | 0.00017 | 0.002696118 |
| VDAC3 | 1.459378735 | 4.154991637 | 0.00064 | 0.006240516 |  | SLC12A5 | -1.267626760 | -4.52703366 | 0.00029 | 0.003817244 |
| YWHAQ | 1.454902422 | 4.804305598 | 0.00016 | 0.002621476 |  | ADRM1 | -1.265539725 | -4.136118494 | 0.00067 | 0.006360379 |
| RPLP0 | 1.454639617 | 5.841510603 | 0.00002 | 0.000787686 |  | RPL13A | -1.265314764 | -5.189264568 | 0.00007 | 0.001567437 |
| CADPS | 1.449210279 | 4.405864719 | 0.00037 | 0.004472813 |  | MVD | -1.264504233 | -5.191518386 | 0.00007 | 0.001567437 |
| CMPK1 | 1.44871477 | 4.660883425 | 0.00021 | 0.003206048 |  | ANXA11 | -1.262168201 | -5.413022887 | 0.00004 | 0.001310669 |
| GPX4 | 1.443700683 | 5.055166965 | 0.00009 | 0.001891895 |  | CHP1 | -1.252360583 | -6.119813449 | 0.00001 | 0.000585773 |
| SNRPA1 | 1.427136044 | 4.098275571 | 0.00104 | 0.008328954 |  | UBL4A | -1.235870468 | -4.57090043 | 0.00026 | 0.003649778 |
| OSBP | 1.408795437 | 5.641017830 | 0.00004 | 0.001310669 |  | EXOC4 | -1.233740637 | -4.022731442 | 0.00086 | 0.007343217 |
| PPP1CC | 1.408543044 | 4.857062821 | 0.00014 | 0.002460345 |  | MAL2 | -1.233397424 | -4.269513213 | 0.00050 | 0.005407751 |
| H2BC13 | 1.405423269 | 4.873607614 | 0.00014 | 0.002426751 |  | PGM2L1 | -1.232454810 | -4.255556845 | 0.00051 | 0.005453681 |
| TPT1 | 1.404601683 | 5.491557047 | 0.00005 | 0.001336234 |  | C11orf68 | -1.231156504 | -5.435836436 | 0.00004 | 0.001291248 |
| EHD3 | 1.40378567 | 5.425187550 | 0.00004 | 0.001308072 |  | PRRT3 | -1.230245658 | -4.297730883 | 0.00061 | 0.006069053 |
| GPHN | 1.397543724 | 4.819885777 | 0.00015 | 0.002573289 |  | SKIV2L | -1.229448984 | -4.797269419 | 0.00016 | 0.002641854 |
| VPS29 | 1.394224481 | 5.331287166 | 0.00008 | 0.001689308 |  | CA11 | -1.229216267 | -3.836068805 | 0.00129 | 0.009572853 |
| MAP1A | 1.387691512 | 5.407351461 | 0.00004 | 0.001310669 |  | DCXR | -1.220464771 | -4.613696826 | 0.00024 | 0.003453217 |
| MACROD1 | 1.38478591 | 4.062312494 | 0.00087 | 0.007457167 |  | CDK18 | -1.214099405 | -4.200531959 | 0.00058 | 0.005912409 |
| SOD2 | 1.381907731 | 4.325692769 | 0.00044 | 0.004997722 |  | ATP5F1A | -1.213878527 | -4.19971342 | 0.00058 | 0.005912409 |
| PLIN3 | 1.374577123 | 4.021863306 | 0.00107 | 0.008426404 |  | RACK1 | -1.212490077 | -4.55979105 | 0.00027 | 0.003692867 |
| DDAH1 | 1.36751446 | 4.090997092 | 0.00074 | 0.006738206 |  | CHD4 | -1.210124706 | -4.113652705 | 0.00089 | 0.007481801 |
| PFN1 | 1.367338032 | 5.323582336 | 0.00005 | 0.001390708 |  | CYTH2 | -1.207454285 | -5.176103014 | 0.00007 | 0.001586723 |
| RALA | 1.363366551 | 4.404838158 | 0.00049 | 0.005335991 |  | NRXN1 | -1.204280934 | -4.24670714 | 0.00052 | 0.005531384 |
| SLC17A7 | 1.350292726 | 4.451487545 | 0.00034 | 0.004185751 |  | KCNA2 | -1.201266458 | -4.137417992 | 0.00067 | 0.00635914 |
| ST13 | 1.349053523 | 4.933648443 | 0.00012 | 0.002284735 |  | WDR91 | -1.200943786 | -4.573171592 | 0.00026 | 0.003645991 |
| GRHPR | 1.348142246 | 5.288622566 | 0.00006 | 0.001436589 |  | ALKBH4 | -1.199817532 | -4.602991139 | 0.00033 | 0.004126976 |
| RAB10 | 1.34800961 | 3.984885007 | 0.00093 | 0.007661005 |  | RPS28 | -1.183125428 | -4.016767066 | 0.00087 | 0.007410309 |
| PPP2CA | 1.34676165 | 4.057455698 | 0.00079 | 0.006985962 |  | ERO1A | -1.182679211 | -4.490374463 | 0.00031 | 0.003961486 |
| NAXE | 1.33590717 | 6.062824241 | 0.00001 | 0.00062177 |  | AP2A1 | -1.181132805 | -4.467691379 | 0.00032 | 0.004109812 |
| ATP5F1C | 1.329761397 | 4.755091397 | 0.00018 | 0.002752457 |  | LINGO1 | -1.179206181 | -4.2432774 | 0.00053 | 0.005559351 |
| FGA | 1.328510497 | 4.263373135 | 0.00051 | 0.005439839 |  | VPS26B | -1.177455701 | -4.100329016 | 0.00072 | 0.006630223 |
| ACAA2 | 1.32204724 | 4.345821306 | 0.00048 | 0.00528192 |  | DPP10 | -1.173562923 | -4.720802029 | 0.00019 | 0.002909505 |
| RAP1GDS1 | 1.313793401 | 3.840492983 | 0.00127 | 0.009529475 |  | RPL7 | -1.173297955 | -5.330599548 | 0.00005 | 0.001390708 |
| DDT | 1.31211412 | 4.966715418 | 0.00011 | 0.002159716 |  | PHF20L1 | -1.172450122 | -4.231279252 | 0.00054 | 0.005627478 |
| NRAS | 1.310495507 | 4.796687593 | 0.00016 | 0.002641854 |  | RPL13 | -1.167651715 | -4.065600272 | 0.00078 | 0.006961461 |
| PRXL2A | 1.308384864 | 4.265465209 | 0.00050 | 0.005428551 |  | GHDC | -1.166086441 | -4.344122789 | 0.00042 | 0.004864929 |
| GUK1 | 1.307432604 | 4.495853917 | 0.00031 | 0.003949728 |  | NCAN | -1.145165442 | -5.101891521 | 0.00008 | 0.001781936 |
| PRNP | 1.300698932 | 4.183597790 | 0.00077 | 0.006899608 |  | PLCB1 | -1.144960530 | -4.724458921 | 0.00019 | 0.002897084 |
| LAP3 | 1.30002936 | 4.279270360 | 0.00049 | 0.005335991 |  | SLC4A10 | -1.142709712 | -3.855025446 | 0.00123 | 0.009359813 |
| IARS2 | 1.295563383 | 4.453135994 | 0.00038 | 0.004550332 |  | TSC2 | -1.142387923 | -4.566157277 | 0.00026 | 0.003654196 |
| SAR1A | 1.295174341 | 4.255321831 | 0.00066 | 0.006354071 |  | ATP9A | -1.137768989 | -4.805503395 | 0.00016 | 0.002621476 |
| FKBP8 | 1.294720345 | 3.870415181 | 0.00119 | 0.009113342 |  | DOCK3 | -1.135783706 | -3.936448436 | 0.00103 | 0.008279656 |
| TPD52L2 | 1.284350174 | 4.689368277 | 0.00020 | 0.003092699 |  | KCTD12 | -1.122314673 | -4.423094577 | 0.00036 | 0.004351519 |
| PCBP2 | 1.278784637 | 4.548937514 | 0.00027 | 0.003732871 |  | PPM1F | -1.120827897 | -4.017800555 | 0.00086 | 0.007408148 |
| HIBADH | 1.274285428 | 5.298840483 | 0.00007 | 0.001562299 |  | INPP4A | -1.116845739 | -4.39535269 | 0.00038 | 0.004532361 |
| CCDC6 | 1.271978628 | 3.897620928 | 0.00112 | 0.008739261 |  | NENF | -1.115353846 | -4.197841425 | 0.00058 | 0.005922725 |
| EEF1A1 | 1.270551073 | 5.745383244 | 0.00002 | 0.000855033 |  | PGRMC2 | -1.113339587 | -4.188954674 | 0.00060 | 0.006010604 |
| MDH1 | 1.269589009 | 4.528810125 | 0.00028 | 0.003817244 |  | GRM2 | -1.106723289 | -3.857021939 | 0.00123 | 0.00935473 |
| NRCAM | 1.267250773 | 3.855747361 | 0.00123 | 0.009359813 |  | PDXP | -1.096326409 | -4.150335584 | 0.00065 | 0.00629011 |
| CRKL | 1.265152719 | 5.116326733 | 0.00008 | 0.001737124 |  | ATG5 | -1.095486502 | -3.983889857 | 0.00093 | 0.007661339 |
| FAHD1 | 1.259393139 | 4.090528099 | 0.00082 | 0.007170037 |  | SOD3 | -1.093656009 | -4.086583172 | 0.00074 | 0.006774969 |
| PPFIA1 | 1.258488985 | 5.513026712 | 0.00004 | 0.001167562 |  | GSS | -1.092965901 | -3.810499507 | 0.00136 | 0.009937033 |
| PFKP | 1.255142957 | 4.947521223 | 0.00012 | 0.002229552 |  | BZW2 | -1.091306741 | -3.988993174 | 0.00114 | 0.008823449 |
| STAT1 | 1.249081363 | 4.501695206 | 0.00030 | 0.00393525 |  | GUCY1A2 | -1.089326960 | -4.795014489 | 0.00016 | 0.002641854 |
| SYT1 | 1.24727046 | 4.630298294 | 0.00023 | 0.003365911 |  | CTBP1 | -1.087774610 | -4.10853099 | 0.00071 | 0.006624421 |
| PPIA | 1.222853252 | 3.825388487 | 0.00132 | 0.009749272 |  | AGRN | -1.083282515 | -4.291963419 | 0.00048 | 0.005241465 |
| NCALD | 1.215970894 | 3.902506311 | 0.00111 | 0.008678074 |  | MRPL19 | -1.080552164 | -4.522785155 | 0.00029 | 0.003840614 |
| LANCL2 | 1.203567714 | 4.058237523 | 0.00079 | 0.006985962 |  | IRGQ | -1.078740958 | -4.05864383 | 0.00079 | 0.006985962 |
| ERI3 | 1.199655503 | 4.388960760 | 0.00069 | 0.006540475 |  | NSFL1C | -1.078360783 | -4.181528241 | 0.00060 | 0.006052329 |
| PAICS | 1.195454198 | 3.924761297 | 0.00106 | 0.008401205 |  | PGAM5 | -1.075143557 | -3.827718603 | 0.00131 | 0.0097162 |
| FN3K | 1.194851322 | 5.373770165 | 0.00005 | 0.001344286 |  | TSN | -1.066642380 | -4.100697343 | 0.00072 | 0.006630223 |
| UBE2N | 1.191680911 | 4.010300233 | 0.00088 | 0.007457167 |  | LSM3 | -1.057516542 | -4.116791514 | 0.00070 | 0.006548261 |
| STIM1 | 1.169765823 | 3.991691314 | 0.00129 | 0.009572853 |  | NDUFAF3 | -1.052295873 | -3.841123709 | 0.00127 | 0.009529475 |
| CCT8 | 1.165762067 | 4.739508905 | 0.00018 | 0.002815261 |  | EIF6 | -1.050641412 | -4.308159721 | 0.00060 | 0.006010604 |
| PAFAH1B1 | 1.154655029 | 5.152715744 | 0.00008 | 0.001649472 |  | PTPRD | -1.045959388 | -4.865451123 | 0.00014 | 0.002449159 |
| INPP1 | 1.150538611 | 3.999048133 | 0.00127 | 0.009524522 |  | CNOT1 | -1.036958744 | -4.173935375 | 0.00061 | 0.006090282 |
| DDX5 | 1.139589224 | 4.764207914 | 0.00017 | 0.002725843 |  | PCDH10 | -1.027766277 | -4.121534972 | 0.00077 | 0.006922089 |
| RAP2A | 1.128224654 | 4.042097667 | 0.00082 | 0.007166236 |  | RPL26 | -1.026954246 | -3.903828116 | 0.00111 | 0.00866855 |
| DNAJC6 | 1.115121715 | 4.125901775 | 0.00068 | 0.006475356 |  | ANKH | -1.010107846 | -4.22040342 | 0.00071 | 0.006630223 |
| RUFY3 | 1.109074913 | 4.689009329 | 0.00020 | 0.003092699 |  | LRP1 | -1.008534095 | -4.043684747 | 0.00082 | 0.007155806 |
| HNRNPR | 1.107651112 | 4.060624602 | 0.00079 | 0.006985962 |  |  |  |  |  |  |
| ALDH2 | 1.103647612 | 4.010823323 | 0.00088 | 0.007457167 |  |  |  |  |  |  |
| TXNDC17 | 1.103554007 | 4.433611512 | 0.00040 | 0.00465155 |  |  |  |  |  |  |
| ECI1 | 1.10131859 | 3.987626333 | 0.00115 | 0.008832619 |  |  |  |  |  |  |
| H1-3 | 1.095383271 | 4.032664868 | 0.00105 | 0.008332101 |  |  |  |  |  |  |
| NDUFV2 | 1.086693782 | 3.811236175 | 0.00136 | 0.009937033 |  |  |  |  |  |  |
| EIF4A2 | 1.085672108 | 4.318590054 | 0.00051 | 0.005442069 |  |  |  |  |  |  |
| SNX3 | 1.082781013 | 5.081197625 | 0.00009 | 0.001834429 |  |  |  |  |  |  |
| PLD3 | 1.073694168 | 4.292923312 | 0.00047 | 0.005241465 |  |  |  |  |  |  |
| RPL11 | 1.071152793 | 4.157749263 | 0.00064 | 0.006240516 |  |  |  |  |  |  |
| UBE2V2 | 1.059443962 | 4.063370129 | 0.00078 | 0.006981108 |  |  |  |  |  |  |
| ACOT7 | 1.056869204 | 4.531872974 | 0.00028 | 0.003814136 |  |  |  |  |  |  |
| ARF3 | 1.051656577 | 4.359358097 | 0.00041 | 0.00474005 |  |  |  |  |  |  |
| ACTB | 1.047954845 | 4.166458265 | 0.00063 | 0.006141705 |  |  |  |  |  |  |
| CAB39L | 1.044289131 | 4.465411837 | 0.00037 | 0.004483765 |  |  |  |  |  |  |
| HSPA8 | 1.037275281 | 4.041020691 | 0.00082 | 0.007168719 |  |  |  |  |  |  |

**Supplementary Table S3**

Antibodies used for immunocytochemistry

| Primary antibodies | Companies | Catalog  number | Host  species | Dilution | Antigen retrieval |
| --- | --- | --- | --- | --- | --- |
| SARS-CoV-2  Nucleoprotein | Sino Biological Inc. | 40143-  R019 | Rabbit | 1:800 | C |
| CD4 | MXB Biotechnology | RMA-0620 | Rabbit | Ready to use | E |
| CD8 | MXB Biotechnology | RMA-0514 | Rabbit | Ready to use | E |
| CD20 | MXB Biotechnology | Kit-0001 | Mouse | Ready to use | E |
| CD3 | MXB Biotechnology | Kit-0003 | Rabbit | Ready to use | E |
| CD14 | Abcam | ab183322 | Rabbit | 1:500 | C |
| CD16 | Abcam | ab246222 | Rabbit | 1:4000 | E |
| CD141 | Abcam | ab109189 | Rabbit | 1:50 | E |
| CD11c | Thermo Fisher | PA5-35326 | Rabbit | 1:100 | E |
| HLA-DR | Abcam | ab92511 | Rabbit | 1:500 | E |
| CCR7 | Abcam | ab253187 | Rabbit | 1:50 | E |
| GFAP | OriGene | ZA-0529 | Rabbit | Ready to use | E |
| IBA-1 | Abcam | ab5076 | Goat | 1:8000 | C |
| IL-4 | Abcam | ab239508 | Mouse | 1:400 | C |
| IL-6 | Abcam | ab6672 | Rabbit | 1:600 | E |
| IL-8 | Bio-Rad | AHP781 | Rabbit | 1:1000 | C |
| IL-12 | Abcam | ab9992 | Goat | 1:1200 | C |
| TGF-β | Proteintech | 21898-1-AP | Rabbit | 1:1000 | C |
| TNF-α | Cell Signaling Technology | 8184S | Rabbit | 1:20 | E |

All antibodies were used for staining on the tissues of COVID-19 brains and controls. C=Citrate buffer (10 mM, pH 6.0); E=EDTA (EDTA 1 mM, Tris 10 mM), pH 9.0.

<insert Table S1 here followed by a page break >

**Supplementary Table S4**

The sequences of primers and probes used in RT-PCR

|  | **Target 1 (open reading frame 1ab, ORF1ab)** | **Target 2 (nucleoprotein, N)** |
| --- | --- | --- |
| Forward | 5'-CCCTGTGGGTTTTACACTTAA-3' | 5'-GGGGAACTTCTCCTGCTAGAAT-3' |
| Reverse | 5'-ACGATTGTGCATCAGCTGA-3' | 5'-CAGACATTTTGCTCTCAAGCTG-3' |
| Probes | 5'-FAM-CCGTCTGCGGTATGTGGAAAGGTTATGG-  BHQ1-3' | 5'-FAM-TTGCTGCTGCTTGACAGATT**-**  TAMRA-3' |
